# Investigation of lignocellulolytic enzymes during different growth phases of *Ganoderma lucidum* strain G0119 using genomic, transcriptomic and secretomic analyses

**DOI:** 10.1371/journal.pone.0198404

**Published:** 2018-05-31

**Authors:** Shuai Zhou, Jingsong Zhang, Fuying Ma, Chuanhong Tang, Qingjiu Tang, Xiaoyu Zhang

**Affiliations:** 1 Key Laboratory of Biophysics of MOE, College of Life Science and Technology, Huazhong University of Science and Technology, Wuhan, People's Republic of China; 2 National Engineering Research Centre of Edible Fungi, Key Laboratory of Applied Mycological Resources and Utilisation, Ministry of Agriculture, Institute of Edible Fungi, Shanghai Academy of Agriculture Sciences, Shanghai, People's Republic of China; Institut National de la Recherche Agronomique, FRANCE

## Abstract

*Ganoderma lucidum* is a medicinal mushroom that is well known for its ability to enhance human health, and products made from this fungus have been highly profitable. The substrate-degrading ability of *G*. *lucidum* could be related to its growth. CAZy proteins were more abundant in its genome than in the other white rot fungi models. Among these CAZy proteins, changes in lignocellulolytic enzymes during growth have not been well studied. Using genomic, transcriptomic and secretomic analyses, this study focuses on the lignocellulolytic enzymes of *G*. *lucidum* strain G0119 to determine which of these degradative enzymes contribute to its growth. From the genome sequencing data, genes belonging to CAZy protein families, especially genes involved in lignocellulose degradation, were investigated. The gene expression, protein abundance and enzymatic activity of lignocellulolytic enzymes in mycelia over a growth cycle were analysed. The overall expression cellulase was higher than that of hemicellulase and lignin-modifying enzymes, particularly during the development of fruiting bodies. The cellulase and hemicellulase abundances and activities increased after the fruiting bodies matured, when basidiospores were produced in massive quantities till the end of the growth cycle. Additionally, the protein abundances of the lignin-modifying enzymes and the expression of their corresponding genes, including laccases and lignin-degrading heme peroxidases, were highest when the mycelia fully spread in the compost bag. Type I cellobiohydrolase was observed to be the most abundant extracellular lignocellulolytic enzyme produced by the *G*. *lucidum* strain G0119. The AA2 family haem peroxidases were the dominant lignin-modifying enzyme expressed during the mycelial growth phase, and several laccases might play roles during the formation of the primordium. This study provides insight into the changes in the lignocellulose degradation ability of *G*. *lucidum* during its growth and will facilitate the discovery of new approaches to accelerate the growth of *G*. *lucidum* in culture.

## Introduction

*Ganoderma lucidum* (Leyss. ex Fr.) Karst, which is also known as “Lingzhi”, is a lamella-less basidiomycetous fungus that belongs to family Polyporaceae [1]. *G*. *lucidum* has been used as a traditional herbal medicine for the treatment of various diseases, including hepatopathy, nephritis, neurasthenia and asthma [2]. As a species of white-rot fungus, *G*. *lucidum* decays plant biomass via secreted enzymes. Similar to other mushrooms with economic value, *G*. *lucidum* is artificially cultivated in logs or compost. Typically, the compost used to cultivate mushrooms contains approximately 60–70% lignocellulose by dry weight [3]. The lignocellulose in the compost is degraded and transformed into the fungal biomass [4, 5] and accounts for 85% of the fruiting body and 45% of the mycelial dry weights [6]. Thus, the substrate-degrading ability of *G*. *lucidum* could be a factor related to its growth.

Lignocellulolytic enzymes are carbohydrate active enzymes (CAZy) that perform important roles in the carbohydrate metabolism of organisms. Lignocellulolytic enzymes are categorized as cellulases, hemicellulases and lignin-modifying enzymes. Cellulases and hemicellulases typically belong to the glycoside hydrolase (GH) family and primarily hydrolyse polysaccharides, such as β-1,4-glucan, xylan, arabinogalactan and mannan, with typical enzymes including endo-β-1,4-glucanase, cellobiohydrolase, β-glucosidase, endo-β-1,4-xylanases, β-1,4-xylosidases, β-galactosidases and β-mannosidases. Common lignin-modifying enzymes primarily include laccases, lignin peroxidases, manganese peroxidases and versatile peroxidases, which belong to the auxiliary activity (AA) protein family [7–9]. Genomic analysis of *G*. *lucidum* has indicated the presence of more CAZy protein genes than other white rot fungi models, including *Phanerochaete chrysosporium* and *Schizophyllum commune* [10, 11]. In addition, transcriptomic and proteomic analyses have identified 120 CAZy proteins and 61 lignocellulose-degrading proteins in *G*. *lucidum*. A comparison of the mycelia and fruiting bodies showed that the GH5, GH31, GH3 and GH16 family protein-encoding genes were present at significantly higher levels in the fruiting bodies, whereas the expression of the fungal oxidative lignin enzymes did not differ [12, 13]. However, these results do not provide detailed information on the changes in lignocellulolytic enzyme expressions during *G*. *lucidum* growth, especially changes in these enzymes secreted into the compost-embedded mycelium [14]. Furthermore, isozymes are often contained in several types of lignocellulose-degrading enzyme, which multiplies the total number of enzymes [15]. However, the enzymes in *G*. *lucidum* that contribute the most to lignocellulose degradation have not been studied. In addition, because the fruiting bodies of *G*. *lucidum* are typically collected after the majority of their basidiospores are ejected (the spores are also collected during the production process), the entire *G*. *lucidum* growth cycle during cultivation generally lasts half a year, which is much longer than the growth cycles of other basidiomycetes [16]. However, whether the expression pattern of lignocellulolytic enzymes during *G*. *lucidum* growth is similar to that in other commercially cultivated mushrooms is unknown.

Based on these considerations, the *G*. *lucidum* strain G0119, which has been commercially cultivated on a large scale in China since 2007 [17], was investigated in this study. To gain more detailed information concerning its lignocellulolytic enzymes, mycelium samples from its compost were assessed by genomic, transcriptomic and secretomic analyses during different growth phases. Our study uncovered valuable information about its lignocellulose-degrading enzymes and the dominant enzymes demonstrating significant changes during the *G*. *lucidum* growth cycle were identified.

## Materials and methods

### Strain, cultivation and sample collection

*G*. *lucidum* G0119 (Hunong Lingzhi No. 1 variety) was obtained from the Institute of Edible Fungi at the Shanghai Academy of Agriculture Sciences, Shanghai, China. The strain was cultured on potato-dextrose agar medium, and a mycelium sample was cultured in potato-dextrose liquid medium for genomic DNA extraction. The culture medium for the fruiting bodies was prepared in a compost bag containing 78% sawdust (a mixture of maple, chestnut and mulberry), 13% wheat bran, 7% corn flour, 1% sucrose and 1% gypsum powder with 60% moisture. Each plastic bag was filled with 600 g (wet weight) of the substrate and capped using a plastic cap with a membrane on top. The diameter and the height of each compost bag was approximately 9 × 15 cm, respectively. After sterilization for two hours at 120 °C and a cool down, the composts were inoculated with mycelium cultured on PDA medium. Mycelial growth proceeded in a culture room with a temperature of 22–25 °C for 2–3 months. During the development of the fruiting bodies, the temperature was controlled at 30–35 °C and the humidity was controlled at 80–95% using water spray.

**Fig 1:**
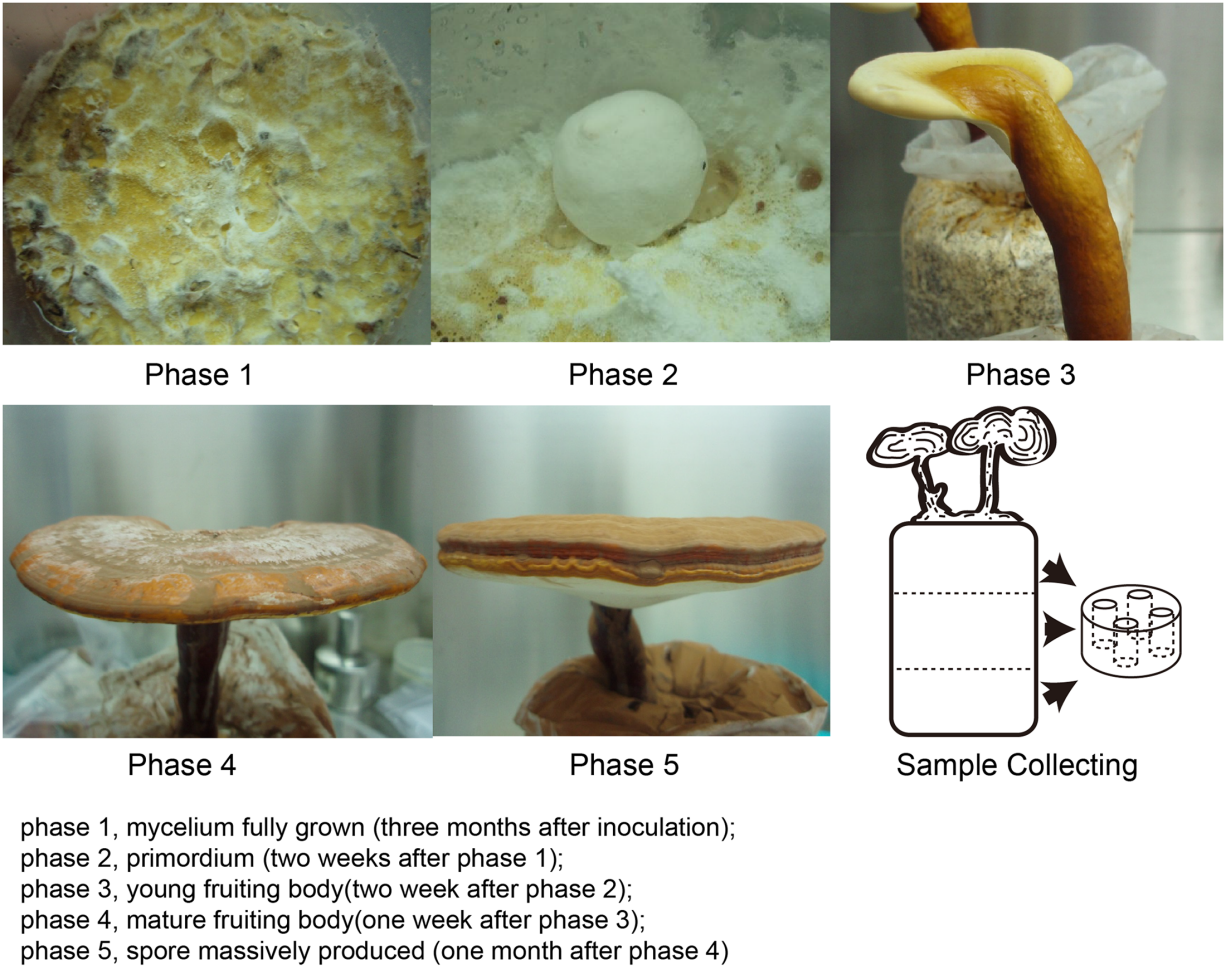
Morphology of the five growth phases of *G*. *lucidum* G0119 and an illustration of sample collection.

The growth cycle of *G*. *lucidum* was divided into 5 typical phases (Fig 1) based on morphology and sample collecting time during each phase: phase 1, mycelium fully grown (three months after inoculation); phase 2, primordium (two weeks after phase 1); phase 3, young fruiting bodies (two weeks after phase 2); phase 4, mature fruiting bodies (one week after phase 3); and phase 5, massive spore production (one month after phase 4). The mycelial biomass and maturity were heterogeneous in different regions of the compost, which could affect the homogeneity of the collected samples [18]. Therefore, each compost bag was divided into an upper, middle and lower layer (the layers were obtained 0–5, 5–10 and 10–15 cm from the top of the compost, respectively) during each growth phase. Mycelial samples together with medium were collected from four uniformly distributed points in each layer of the compost substrate. Samples from two biological replicates at each growth phase were prepared for RNA-seq and label-free analysis. All collected samples were first frozen in liquid nitrogen and then preserved at -80 °C.

### Genome sequencing and functional annotation of lignocellulolytic enzymes

Genomic DNA was extracted from the *G*. *lucidum* mycelia samples using the cetyltrimethylammonium bromide (CTAB) method as follows. A total of 100 mg of freeze-dried mycelium was ground in a mortar which was sterilized at 121 °C for 25 min and precooled to -80 °C, and then mixed with 1.4 mL of CTAB buffer [2% CTAB, first sterilization at 121 °C for 25 min, followed by the addition of β-mercaptoethanol at 0.5% and proteinase K at 0.35 mg/mL] and 70 μL of 10% sterilized SDS solution. After incubation at 65 °C for 30 min, 600 mL of a phenol: chloroform: isoamyl alcohol mixture (25:24:1, v/v/v) was added, and the mixture was allowed to rest at room temperature for 3–5 min. After centrifugation at 12,000 x g at 4 °C for 10 min, the supernatant was mixed with 700 μL of a chloroform: isoamyl alcohol mixture (24:1, v/v). After centrifugation at 12,000 x g at 4 °C for 8 min, the supernatant was mixed with three volumes of 100% ethanol (precooled to -20 °C). After incubating at -20 °C for 10–30 min and then centrifuging at 12,000 x g at 4 °C for 10 min, the precipitate was washed with 1 mL of 100% ethanol and centrifuged again. After air-drying, the precipitate was dissolved in 50 μL of TE buffer (pH 8.0), and 1 μL of RNase (10 mg/mL, Takara Bio, Shiga, Japan) was added, followed by incubation at 37 °C for 1 hour to remove the RNA. The extracted DNA was quantified and assessed for quality using a Nanodrop (Thermo Scientific, MA, USA) and agarose gel electrophoresis, respectively. The genomic DNA was sequenced on a Roche 454 GS FLX (Roche, CT, USA) and assembled on an Illumina Solexa GAIIx (Illumina, CA, USA). The coverage depth was 15X. During scaffold assembly, gene models were predicted using GeneMark (http://exon.gatech.edu/GeneMark/), Augustus (http://augustus.gobics.de/) and Fgenesh (http://www.softberry.com) and annotated with the KEGG (http://www.genome.jp/kegg/), nr (https://ftp.ncbi.nlm.nih.gov/blast/db/FASTA/) and UniProt databases (http://www.uniprot.org/). Then, the gene models were transcribed into amino acid sequences using the RevTrans 1.4 Server [19], and the CAZy proteins in these gene models were annotated with the CAZyme database using Hmmer 3.0 and HMM data from dbCAN [20]. Signal peptides and transmembrane helices in proteins were predicted with SignalP 4.0 and TMHMM 2.0, respectively [21, 22].

### RNA extraction and RNA-seq

One hundred milligram of each sample was ground in liquid nitrogen and then mixed with 1.5 mL of the TRIzol reagent (Invitrogen, CA, USA). After incubating for 5 min at room temperature, the mixture was centrifuged at 12000 x g and 4 °C for 5 min. Next, 1 mL of the supernatant was transferred to a new tube, mixed with 0.2 mL of chloroform and vortexed for 15 seconds. The tubes were incubated at room temperature for 3 min. After centrifuging at 12,000 x g and 4 °C for 15 min, 0.5 mL of the aqueous phase was transferred to a new tube, 0.5 mL of isopropanol was added, and the tube was vortexed to precipitate the RNA. After incubating the tube at room temperature for more than 30 min and centrifuging at 12,000 x g and 4 °C for 10 min, the supernatant was discarded. Then, 1 mL of 75% ethanol was added to the tube, which was centrifuged at 6,000 x at 4 °C for 5 min. This procedure was repeated twice. The remaining ethanol was removed using a pipette, and the RNA was dissolved in 80μL of RNase-free water. The RNA quantity and quality were determined using a Nanodrop and by performing agarose gel electrophoresis, respectively. A cDNA library was constructed according to the Illumina protocol, which included the mRNA purification and fragmentation, the synthesis of first and second strand cDNA, end repair, the adenylation of the 3’ ends and adapter ligation steps. The DNA fragments were enriched by PCR using a PCR primer cocktail and PCR master mix. The libraries were quantified using a Qubit (Invitrogen, CA, USA), and clustering was accomplished with cBot (Illumina). Sample libraries were sequenced using a HiSeq 3000 (Illumina).

Adaptor sequences and low-quality reads among the cDNA sequences were filtered at the 3’ end of the raw reads using Trim Galore 0.4.0, and FastQC 0.11.3 was used for data quality control. Clean reads were obtained and mapped to the genome of *G*. *lucidum* G0119. Gene expression levels were calculated with the FPKM method, using RSEM with default parameters [23]. A clustering analysis of gene expression was performed using the statistical platform R with the pheatmap package. Differentially expressed genes (DEGs) were identified using the DESeq package by calculating log2 (FPKM+1). Genes with fold changes in expression of greater than 2 and *P*-value less than 0.05 were treated as DEGs. Fisher’s exact test was used to calculate *P*-values, and the Benjamini-Hochberg correction was applied.

### Label-free analysis of extracellular proteins

Freeze-dried samples (two replicates per sample) were ground with dry ice, and 10 g of each sample was extracted with 100 mL of water in a flask. After incubating on a shaker set at 200 rpm and 4 °C for 1 hour to dissolve the extracellular proteins, the solution was centrifuged at 10,000 x g for 15 min. Then, 10 mL of the supernatant was transferred to a new tube and vortexed with a 5-fold volume of a trichloroacetic acid (TCA) and acetone mixture (1:9, v/v). The mixture was placed at -20 °C for at least 4 hours and then centrifuged at 6,000 g for 40 min at 4 °C. The precipitate was then washed three times with pre-cooled acetone and air dried in a fume hood. The protein sample was quantified with a BCA Protein Assay Kit (Bio-Rad, CA, USA) [24], and quality was assessed by performing SDS-PAGE and Coomassie Blue R-250 staining. The protein samples were stored at -80 °C. Based on the protein concentration data, a calculated volume of each sample was transferred to ensure that the total protein amounts in the subsequent procedures were equal. After samples were digested with trypsin (Promega, WI, USA), the resultant peptides in each sample were desalted on C18 cartridges (Empore^™^ SPE Cartridges C18 (standard density), bed I.D.: 7 mm, volume: 3 mL; Sigma-Aldrich, MO, USA), concentrated by vacuum centrifugation and reconstituted in 40 μL of 0.1% (v/v) formic acid. The peptide content was estimated using a BCA assay kit.

Samples were assessed using a nanoLC-MS/MS system equipped with an Easy nLC high-performance liquid chromatography (HPLC) and a Q Exactive mass spectrometer (MS) (Thermo Scientific, CA, USA). Each fraction was first injected for HPLC separation. The peptide mixture was loaded onto a reverse-phase trap column (Thermo Scientific Acclaim PepMap100, 100 μm × 2 cm, nanoViper C18) connected to a C18 reversed-phase analytical column (Thermo Scientific Easy Column, 10 cm long, 75 μm inner diameter, and a 3 μm particle size) in buffer A (0.1% formic acid) and separated using a linear gradient of buffer B (84% acetonitrile and 0.1% formic acid) at a flow rate of 300 nL/min. The linear gradient consisted of 0–35% buffer B for 50 min, 35–100% buffer B for 5 min, and a hold at 100% buffer B for 5 min. After HPLC separation, the samples were analysed using the Q Exactive mass spectrometer. The total analysis time was 60 min, and the mass spectrometer was operated in positive ion mode. MS data were acquired using a data-dependent top-10 method that dynamically selected the most abundant precursor ions from the survey scan (300–1800 m/z) for high-energy collisional dissociation (HCD) fragmentation. The automatic gain control (AGC) target was set to 3e6, and the maximum injection time was 10 ms. The dynamic exclusion duration was 40.0 s. Survey scans were acquired at a resolution of 70,000 at m/z 200 and the resolution for HCD spectra was set to 17,500 at m/z 200, with an isolation width of 2 m/z. The normalized collision energy was 30 eV, and the underfill ratio was defined as 0.1%. The quality of the MS data was assessed using Andromeda [25] and analysed using MaxQuant v1.3.0.5 (Max Planck Institute of Biochemistry, Martinsried, Germany) [26] by mapping the data to the G0119 protein database. Protein abundance was quantified by determining and normalizing the intensity using the MaxLFQ method [27]. Proteins with fold changes in abundance greater than 2 and *P*-values less than 0.05 were treated as differentially abundant proteins.

### Enzyme activity tests for lignocellulolytic enzymes among secreted proteins

Freeze-dried samples (three replicates per sample) were ground, and 5 g of each sample was extracted with 50 mL of 100 mM sodium acetate buffer (pH 4.5) in a shaker set at 100 rpm and 4 °C for 2 hours. Enzyme solutions were obtained using suction filtration. The protein concentration in the solutions were determined using the Bradford method. Endoglucanase enzyme activity was assayed using an endo-cellulase kit (Megazyme, Ireland) based on the GellG3 method [28]. Cellobiohydrolase, β-glucosidase and β-xylosidase activities were assayed using the reaction substrates 4-nitrophenyl-β-cellobioside (PNPC), 4-nitrophenyl-β-glucoside (PNPG), and 4-nitrophenyl-β-xyloside (PNPX), respectively, and measuring the absorption of 4-nitrophenol at 400 nm [18]. The laccase and peroxidase activities were measured by detecting the oxidation of 2,2′-azinobis-3-ethylbenzothiazoline-6-sulfonic acid (ABTS) at 420 nm [29] and the oxidation of guaiacol at 465 nm [30], respectively. All enzyme activities were calculated as units/mg protein of the sample.

### Lignin, cellulose and hemicellulose determinations

Five hundred milligrams of each ground sample (three replicates per sample) was extracted with 10 mL of sodium acetate buffer (18.6 g/L of EDTA, 6.8 g/L of sodium borate, 30 g/L of lauryl sodium sulphate, 4.56 g/L of Na_2_HPO_4_ and 10 mL/L of glycol ether, pH 6.9–7.1) in a thermo shaker at 500 rpm and 100 °C for 1 hour to remove neutral soluble substances. After centrifuging at 3500 x g for 10 min, the supernatants were discarded; then, the precipitates were washed with 10 mL of distilled water three times and centrifuged under the same conditions. The precipitates were dried in an RVC 2–25 CD plus vacuum centrifuge concentrator (Martin Christ, Osterode, Germany) at 70 °C for 3 hours. Then, the precipitates were hydrolysed with 10 mL of 2 M HCl in a thermo shaker at 500 rpm and 100 °C for 2 hours. After centrifuging under the same conditions, the supernatants were collected, and the precipitates were washed with distilled water and centrifuged under the same conditions. The precipitates were dried using a vacuum centrifuge concentrator as described above. Next, 1 mL of 72% (w/w) H_2_SO_4_ was added to each dried precipitate and kept at room temperature for 1 hour. Then, 9 mL of distilled water was added to each sample and hydrolysed in a thermo shaker at 500 rpm and 100 °C for 3 hours. After centrifuging, the supernatants were collected for the monosaccharide determinations, and the precipitates were filtered using P2 glass crucibles (VELP Scientifica, Usmate, Italy) and washed with distilled water three times. The precipitates in the crucibles were dried at 105 °C for 4 hours. After weighing, the precipitates were kept in a muffle furnace at 525 °C for 5 hours to determine the ash weight. Lignin was calculated as the dry weight of precipitate minus the ash weight. The monosaccharide concentrations in the HCl and H_2_SO_4_ hydrolysis steps were determined using the ICS5000+ HPAEC-PAD system (Thermo Scientific, CA, USA) with a CarboPac PA20 column (150 x 3 mm, Dionex, CA, USA). The column temperature was 30 °C, and the mobile phase was 2 mM NaOH at a flow rate of 0.40 mL/min. The external standard monosaccharides included fucose, rhamnose, arabinose, galactose, glucose, mannose, xylose, fructose, glucuronic acid (GlcU) and galacturonic acid (GalU) (Sigma-Aldrich, MO, USA). The glucose content was calculated as the cellulose content (w/w). The total arabinose, galactose, mannose and xylose contents were calculated as the hemicellulose content (w/w).

## Results

### Lignocellulolytic enzymes encoded in the G0119 genome

The *G*. *lucidum* G0119 genome (accession number: PRJNA406843) consisted of 41.49 Mb and was assembled into 2264 contigs that formed 348 scaffolds. GeneMark, Augustus and Fgenesh predicted 10,525, 12,688 and 10,683 complete gene models, respectively. After annotation using the KEGG, nr and Uniport databases, 11,123 gene models were selected out.

Mapping the CAZyme data identified and classified 565 CAZy genes in the G0119 genome, including 262 genes from 53 GH families, 33 genes from 9 CBM families, 96 genes from 9 AA families, 72 genes from 30 glycosyltransferase (GT) families, 91 genes from 10 carbohydrate esterase (CE) families and 11 genes from 4 polysaccharide lyase (PL) families. The distribution of CAZy protein family members in *G*. *lucidum* G0119 is shown in S1 Table.

Of the CAZy coding genes in the G0119 genome, 92 genes were identified as putative enzymes involved in lignocellulose degradation based on their functional annotation, accounting for 16% of the total CAZy genes identified. Gene IDs and CAZy family assignments are listed in S2 Table. Cellulases were assumed to be primarily represented by the GH1, GH3, GH5, GH7, GH12, and AA9 (formerly GH61) families, and hemicellulases were primarily represented by the GH2, GH3, GH10, GH35 and GH51 families. Lignin-modifying enzymes were primarily represented by the AA1 and AA2 families. Most lignocellulolytic enzymes contained a signal peptide of approximately 20 amino acids in length, suggesting that they are secreted proteins. Among them, 6 laccases and 1 endoglucanase were secreted proteins that contained an approximately 22 amino acid transmembrane domain for, and their major protein chains were extracellular.

### RNA-seq analysis of gene expression during G0119 growth

The clean reads and bases from RNA-seq accounted for over 92 and 83% of the raw data, respectively. By mapping the clean reads to the G0119 genome, 10,316 genes were identified among the mRNAs, accounting for 93% of the total genes. Five hundred and sixty-three CAZy genes were expressed in samples obtained during the 5 growth phases. One cellobiohydrolase gene from the GH6 family and 1 beta-galactosidase gene from the GH35 family were not expressed. The clustering analysis of CAZy gene expression is shown in S1 Fig. Generally, the expression level at phases 3 and 4 were similar. Based on the expression levels, these genes were divided into four groups. Group 3 showed the highest expression levels and comprised 87 genes, 10 of which were involved in lignocellulose degradation, including 6 endo-β-1,4-glucanase genes from the GH5, GH12 and AA9 (formerly GH61) families, 1 cellobiohydrolase (CBH) gene from the GH6 family, 2 β-glucosidase genes from the GH3 family and 1 α-N-arabinofuranosidase gene from the GH51 family. Additional CAZy genes in this group were involved in the degradation of β-1,3-glucan and starch and the synthesis of trehalose, glycogen and chitin (S3 Table). The expression levels of groups 4, 2 and 1 were observed to decrease in this order.

**Fig 2:**
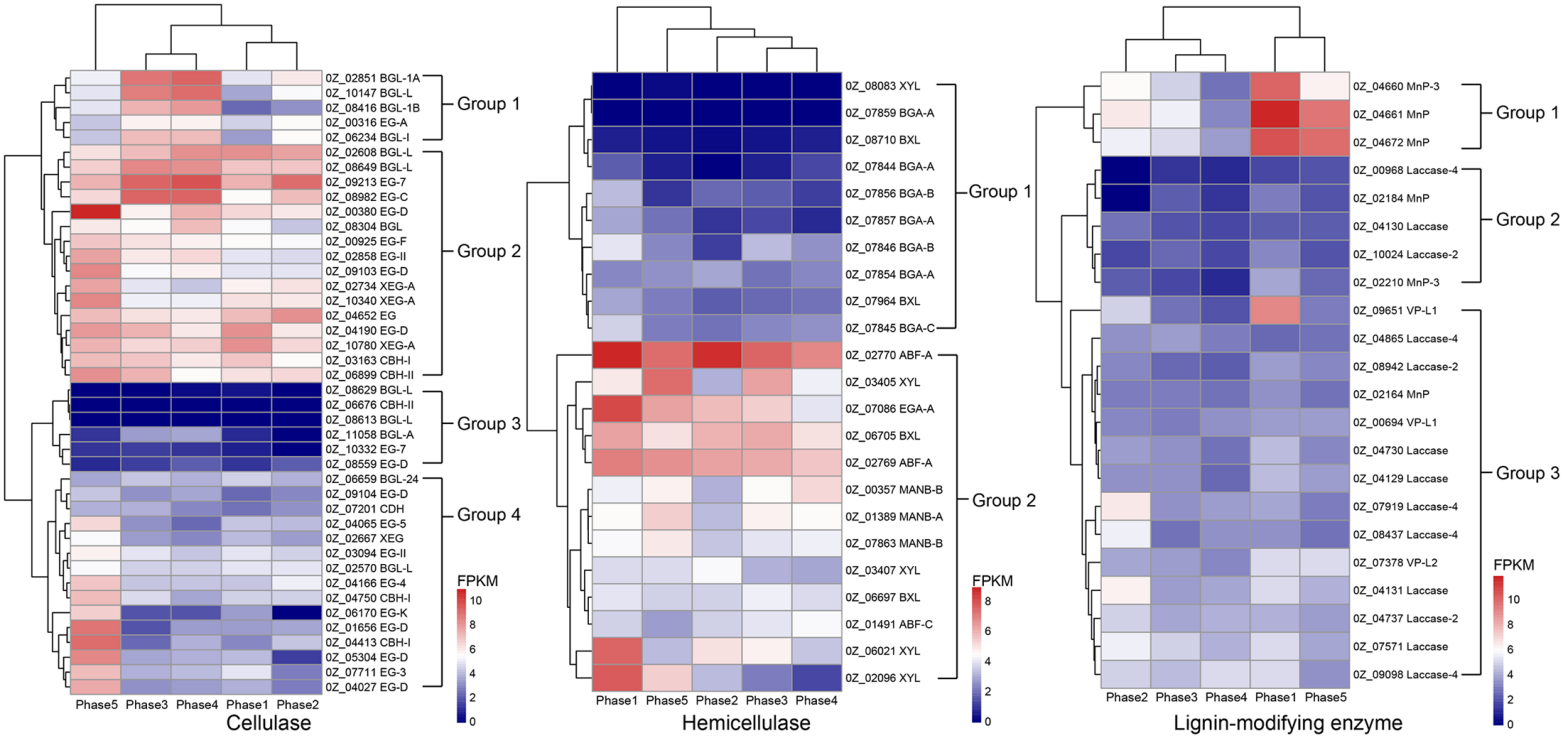
Changes in the expression of lignocellulolytic enzyme-coding genes during *G*. *lucidum* G0119 growth.

The observed changes in the expression of lignocellulolytic enzyme-coding genes are shown in Fig 2. The expression levels of the cellulase-encoding genes were highest in phase 5 among all the phases. The cellulase-encoding genes could be divided into four groups based on their expression levels. The genes in group 2 showed the highest expression during growth and comprised 11 endoglucanase genes from the GH5, GH12, AA9 and CBM1 families, 2 cellobiohydrolase genes from the GH6 and GH7 families, and 4 β-glucosidase genes from the GH3 family. The expression of hemicellulase-encoding genes was briefly increased in phase 1, and these genes were divided into two groups based on their expression levels. In group 2, 5 genes were highly expressed during growth, including 1 xylosidase gene from the GH3 family, 2 arabinofuranosidase genes from the GH51 family, 1 xylanase gene from the GH10 family and 1 endogalactosidase gene from the GH53 family. The expression of genes encoding lignin-modifying enzymes was increased in phases 1 and 5, and these genes were divided into 3 groups based on their expression levels. The highest expression levels were observed in group 1, which contained 3 AA2 haem peroxidase genes that were highly expressed in phases 1 and 5. In group 2, 1 AA2 haem peroxidase gene was highly expressed in phase 1, whereas 5 AA1 laccase genes and 2 AA2 haem peroxidase genes were highly expressed in phase 2. The remainder of the genes in group 2 and the genes in group 3 were expressed at low levels and consisted of genes encoding 7 AA1 laccases and 4 AA2 haem peroxidases.

The significance of the differences in the expression levels of these genes between adjacent growth phases was analysed. DEGs encoding lignocellulolytic enzymes and their significance are shown in Table 1. From phase 1 to phase 2, 1 β-glucosidase gene from the GH3 family and 1 laccase gene from the AA1 family were upregulated by 2.30- and 2.75-fold, respectively. Other genes were downregulated with even higher fold changes, including 1 endoglucanase-encoding gene from the GH45 family (5.19-fold), 2 endoxylanase-encoding genes from the GH10 family (2.51- and 4.52-fold), 1 β-galactosidase-encoding gene from the GH35 family (3.56-fold), 4 haem peroxidase-encoding genes from the AA2 family (5.30-, 5.02-, 3.93- and 4.37-fold). From phase 2 to phase 3, genes encoding 3 β-glucosidases from the GH1 and GH3 families were upregulated by 4.52-, 3.39- and 2.90-fold. Genes encoding 1 endoxylanase from the GH10 family, and 1 β-galactosidase gene from the GH35 family were upregulated by 3.32- and 2.80-fold, respectively. Genes encoding 2 laccases from the AA1 family were downregulated by 3.09- and 2.74-fold. The expression of the heme peroxidase-encoding gene 0Z_04660 was downregulated from phase 3 to phase 4 by 2.27-fold. From phase 4 to phase 5, 20 of 25 DEGs were upregulated. Among them, 11 of 12 endoglucanase-encoding DEGs from the GH5, GH12, GH45, GH74, GH131 and AA9 families were upregulated by fold changes that ranged from 2.59–5.68. Additionally, 3 CBH-encoding genes from the GH6 and GH7 families were upregulated by 5.44-, 3.59- and 2.59-fold, 2 endoxylanase-encoding genes from the GH10 family were upregulated by 3.30- and 4.85-fold, 1 endogalactanase-encoding gene from the GH53 family was upregulated by 2.47-fold, and 3 haem peroxidase-encoding genes from the AA2 family were upregulated by 6.60-, 6.30-, and 3.71-fold. The downregulated DEGs consisted of 1 endoglucanase-encoding gene from the GH5 family (2.76-fold) and 4 β-glucosidase-encoding genes from the GH1 and GH3 families by fold changes ranging from 2.87–4.35.

**Table 1:** Changes in the expression of lignocellulolytic enzyme-encoding genes between *G*. *lucidum* G0119 growth phases.

Gene ID	Function	Phase 2/Phase 1		Phase 3/Phase 2		Phase 4/Phase 3		Phase 5/Phase 4	
		Fold change	P value	Fold change	P value	Fold change	P value	Fold change	P value
0Z_04166	EG-4	1.01	0.39	-0.97	0.43	0.27	0.81	2.64	0.02
0Z_07711	EG-3	-2.14	0.08	1.19	0.38	0.28	0.80	3.30	0.00
0Z_06170	EG-K	-5.19	0.01	3.40	0.23	-0.02	1.00	5.39	0.00
0Z_08982	EG-C	1.74	0.17	2.11	0.12	0.18	0.88	-2.76	0.03
0Z_00380	EG-D	-0.52	0.68	-0.19	0.85	1.62	0.15	3.77	0.00
0Z_01656	EG-D	0.32	0.81	-2.23	0.09	2.04	0.07	5.68	0.00
0Z_04027	EG-D	-1.12	0.42	0.32	0.91	0.59	0.62	4.25	0.00
0Z_05304	EG-D	-2.98	0.04	2.59	0.09	0.32	0.78	4.77	0.00
0Z_09103	EG-D	0.01	0.99	0.55	0.69	0.53	0.62	2.73	0.01
0Z_02734	XEG-A	0.44	0.69	-1.41	0.25	-0.43	0.69	3.48	0.00
0Z_10340	XEG-A	-0.29	0.81	-0.77	0.50	0.18	0.87	3.19	0.00
0Z_02667	XEG	-0.47	0.69	-0.04	0.94	-0.47	0.67	2.59	0.02
0Z_06899	CBH-II	0.32	0.77	0.83	0.52	-1.54	0.15	2.59	0.02
0Z_04413	CBH-I	1.56	0.18	-2.22	0.08	1.82	0.10	5.44	0.00
0Z_04750	CBH-I	-0.01	1.00	0.16	0.94	-0.83	0.44	3.55	0.00
0Z_02851	BGL-1A	1.23	0.31	2.90	0.03	0.63	0.58	-4.35	0.00
0Z_08416	BGL-1B	0.92	0.44	4.52	0.00	0.65	0.54	-3.12	0.01
0Z_06234	BGL-I	2.30	0.05	1.47	0.23	0.16	0.88	-2.87	0.01
0Z_10147	BGL-I	1.77	0.19	3.39	0.02	0.52	0.67	-4.11	0.00
0Z_06021	XYL	-2.51	0.03	-0.35	0.75	-1.18	0.28	-0.28	0.81
0Z_03405	XYL	-1.91	0.10	3.32	0.01	-1.90	0.08	3.30	0.00
0Z_02096	XYL	-4.52	0.00	-1.17	0.37	-1.26	0.30	4.85	0.00
0Z_07086	EGA-A	-2.32	0.04	-0.38	0.72	-1.37	0.20	2.47	0.02
0Z_07846	BGA-B	-3.56	0.01	2.80	0.05	-0.65	0.58	-0.22	0.85
0Z_07919	LACC-4	2.75	0.02	-3.09	0.02	0.32	0.77	-0.69	0.53
0Z_04131	LACC	1.48	0.20	-2.74	0.03	0.33	0.76	0.34	0.76
0Z_04661	MnP	-5.30	0.00	-1.10	0.46	-2.24	0.11	6.60	0.00
0Z_04672	MnP	-5.02	0.00	-0.47	0.71	-1.24	0.32	6.30	0.00
0Z_04660	MnP-3	-3.93	0.00	-1.23	0.32	-2.27	0.05	3.71	0.00
0Z_09651	VP-L1	-4.37	0.00	-2.34	0.06	-0.72	0.53	1.22	0.27

Note: Values with a fold change of greater than 2 and a *P*-value lower than 0.05 are shaded grey.

### Label-free analysis of extracellular protein abundance during *G*. *lucidum* G0119 growth

A total of 926 proteins were identified as extracellular proteins across the growth cycle and accounted for 9% of the transcriptome. CAZy proteins were the most abundant, particularly the CBH isozyme 0Z_04413 from the GH7 family (S2 Fig). The protein abundances of 1 endopolygalacturonase from the GH28 family (0Z_00874), 1 α-galactosidase from the GH27 family (0Z_02552), 3 CBHs from the GH6 and GH7 families (0Z_04750, 0Z_03163, and 0Z_06899), 1 xyloglucanase from the GH74 family (0Z_02667) and 1 endoxylanase from the GH10 family (0Z_03405) were lower in that order. Among these proteins, 0Z_04750 and 0Z_00874 were the most abundant in phase 2 while 0Z_02552 peaked in phase 1. The abundances of the remaining 5 proteins were all highest in phase 5. In general, the abundance of lignin-modifying enzymes was much lower than that of cellulase and hemicellulose enzymes.

**Fig 3:**
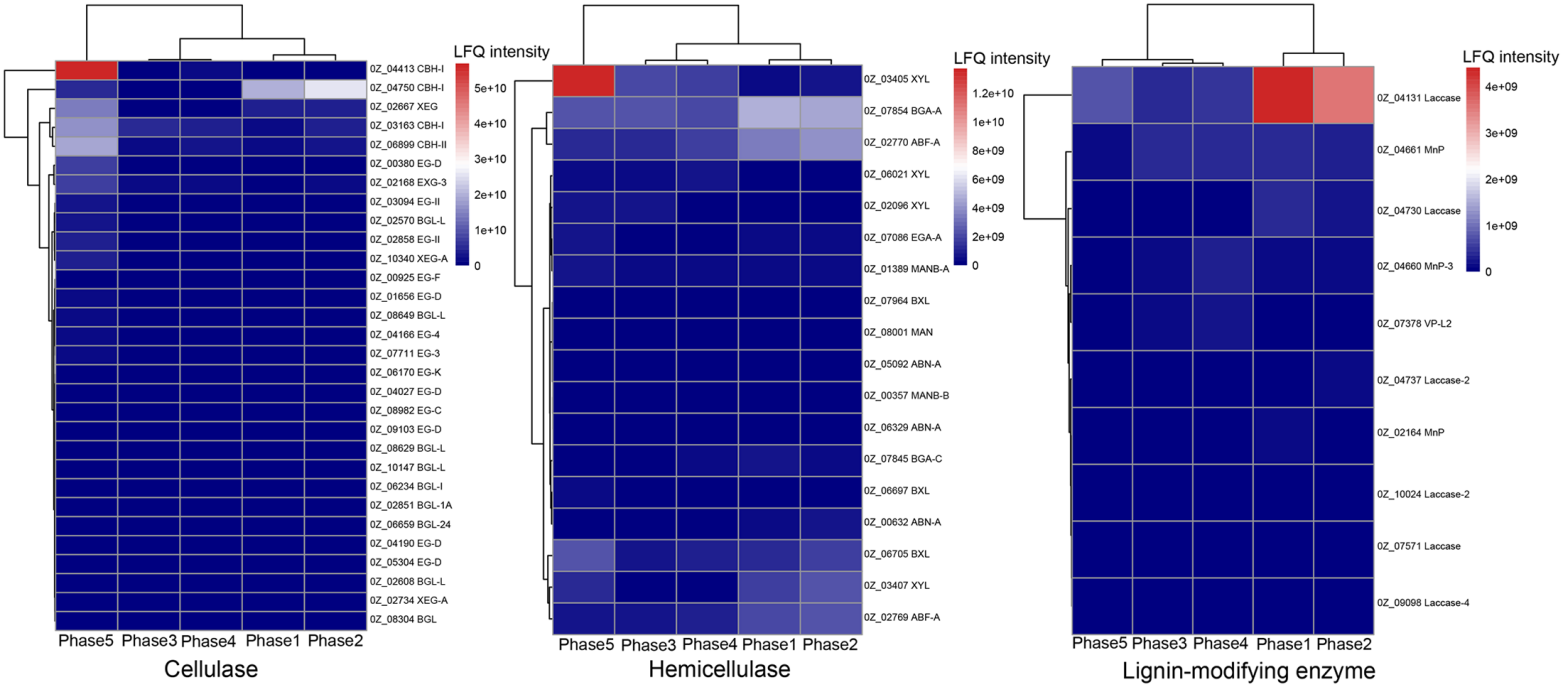
Changes in the protein abundance of lignocellulolytic enzymes during *G*. *lucidum* G0119 growth.

Changes in the abundance of lignocellulolytic enzymes during G0119 growth are shown in Fig 3. The most abundant cellulases were CBH 0Z_04413, 0Z_04750, 0Z_03163, 0Z_06899 and the xyloglucanase 0Z_02667. The most abundant hemicellulase proteins were xylanase 0Z_03405, β-galactosidase 0Z_07854 and arabinofuranosidase 0Z_02770; 0Z_03405 was highly expressed in phase 5, while 0Z_07854 and 0Z_02770 were highly expressed during phases 1 and 2. The lignin-modifying enzyme with the highest abundance was laccase 0Z_04131, which was highly expressed in phases 1 and 2.

Differences in protein abundance between adjacent growth phases were assessed for significance, and proteins with a fold change in abundance of greater than 2.0 and a *P*-value less than 0.05 were considered differentially abundant proteins. These lignocellulolytic enzymes that were differentially abundant between phases are shown in Table 2. The fold changes of the differentially abundant proteins were mostly higher than those observed in gene expression. One heme peroxidase from the AA2 family was downregulated by 3.13-fold from phase 1 to phase 2. From phase 2 to phase 3, 1 CBH from the GH7 family and 1 endoxylanase from the GH10 family were downregulated by 50.00- and 33.33-folds. From phase 3 to phase 4, 1 endoglucanase from the GH12 family was upregulated by 4.03-fold, and 1 endoxylanase from the GH10 family was downregulated by 4.76-fold. From phase 4 to phase 5, 15 out of 16 differentially abundant proteins were upregulated, including 10 endoglucanases from the AA9, GH5, GH12, GH45, GH74, GH131 and CBM1 families by fold changes ranging from 8.13–47.37, 2 CBHs from the GH7 family by 40.41- and 13.31-fold, 1 β-glucosidase from the GH3 family by 14.84-fold, and 2 endoxylanases from the GH10 family by 7.82- and 21.36-fold. The only differentially abundant proteins that was downregulated was a haem peroxidase from the AA2 family by 10.37-fold.

**Table 2:** Differences in the abundance of lignocellulolytic enzymes among *G*. *lucidum* G0119 growth phases.

Protein ID	Function	Phase2/Phase1		Phase3/Phase2		Phase4/Phase3		Phase5/Phase4	
		Fold change	P value	Fold change	P value	Fold change	P value	Fold change	P value
0Z_00925	EG-F	1.85	0.31	-1.89	0.61	1.15	0.69	8.40	0.03
0Z_04166	EG-4	1.51	0.53	-2.78	0.46	1.23	0.57	13.95	0.01
0Z_07711	EG-3	1.50	0.54	-2.78	0.45	1.04	0.87	14.48	0.01
0Z_06170	EG-K	NA	NA	1.59	0.84	-1.06	0.95	39.14	0.00
0Z_00380	EG-D	1.14	0.93	-1.85	0.61	1.12	0.72	47.37	0.00
0Z_01656	EG-D	1.00	0.88	1.05	0.92	2.18	0.06	14.28	0.01
0Z_04027	EG-D	-1.09	0.74	-1.28	0.78	1.88	0.13	27.62	0.00
0Z_05304	EG-D	NA	NA	NA	NA	NA	NA	27.11	0.00
0Z_10340	XEG	1.84	0.32	-1.52	0.70	4.03	0.00	8.13	0.03
0Z_02667	XEG	-1.05	0.79	-16.67	0.06	1.82	0.15	32.55	0.00
0Z_04413	CBH-I	1.19	0.86	-1.22	0.80	1.96	0.10	40.41	0.00
0Z_04750	CBH-I	1.34	0.69	-50.00	0.02	-1.54	0.35	13.31	0.01
0Z_02570	BGL-L	1.03	0.92	-1.25	0.79	-1.08	0.94	14.84	0.01
0Z_03405	XYL	2.67	0.09	2.42	0.58	-1.14	0.83	7.82	0.04
0Z_02096	XYL	1.47	0.56	3.21	0.43	-4.76	0.00	4.69	0.10
0Z_03407	XYL	1.40	0.63	-33.33	0.02	-1.22	0.71	21.36	0.00
0Z_02164	VP/MnP	-3.13	0.01	NA	NA	NA	NA	NA	NA
0Z_04660	MnP-3	1.16	0.90	1.43	0.91	1.71	0.19	-10.37	0.01

Note: Values with a fold change in abundance of greater than 2 and a *P*-value lower than 0.05 are shaded grey. NA = Not available

To learn more about the relationship between the expression and abundance of lignocellulolytic enzymes, this isozyme series was classified into 9 groups based on function and compared individually. The results are shown in S3 Fig. Ninety-five enzyme-encoding genes were detected in the transcriptome, of which 61 enzymes were detected in the secreted protein samples. Possible reasons for this discrepancy are that some genes without signal peptides may be primarily distributed in the cytoplasm. The XEG 0Z_10780, ED-D 0Z_08559, BGL-1B 0Z_08416, BXL 0Z_08710, BGA-B 0Z_07856 and MANB-B 0Z_07863 were such cases. Additionally, some genes distributed in the cytomembrane may also not be detected in the secreted protein samples, such as EG-A 0Z_00316. Sample loss during preparation may also have occurred. The brief expression of genes encoding endoglucanases, CBH (an exoglucanase) and mannosidases increased from phase 1 to phase 5, and their overall protein abundances changed accordingly. Half of the arabinosidases identified in the *G*. *lucidum* G0119 genome were not detected in the protein samples. Most laccases and peroxidases were more highly expressed during phases 1 or 2, while only 2 isozymes had similar changes in protein abundance. One or two isozymes in each group exhibited a higher protein abundance than the others. In addition, some genes showed expression changes that differed from their corresponding changes in protein abundance, possibly due to factors such as posttranscriptional regulation, which was also found in *Schizophyllum commune* [31], rates of mRNA transcription and protein translation and protein stability, as was discussed previously for *A*. *bisporus* [32].

### Activity of secreted lignocellulolytic enzymes during G0119 growth

**Fig 4:**
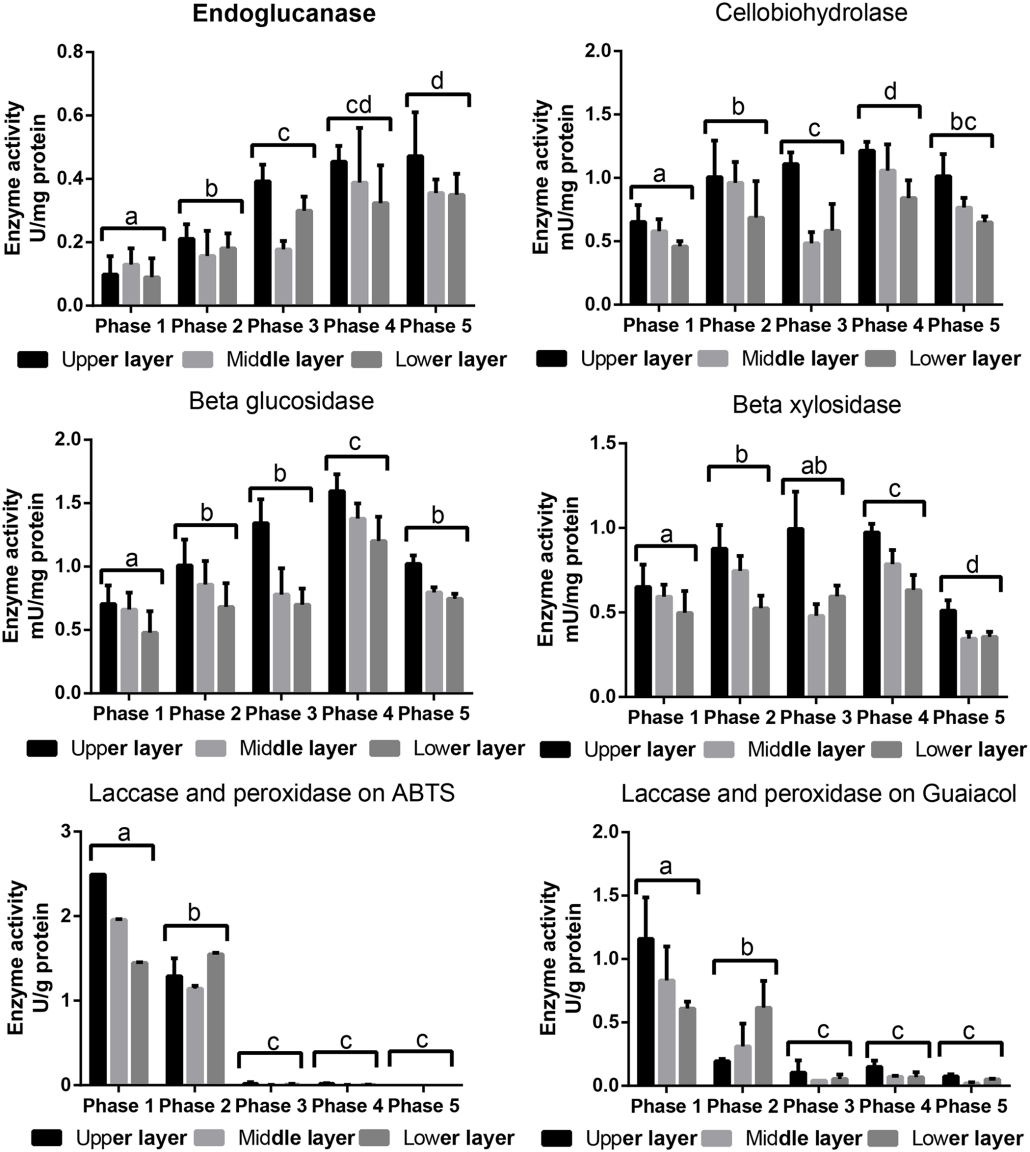
Lignocellulolytic enzyme activities during the 5 *G*. *lucidum* G0119 growth phases.

The protein concentrations obtained from the enzyme activity test are presented in S4 Fig. The protein concentrations increased during growth, with the concentrations at phase 5 2 to 3 times higher than those at the other phases. The activities of the lignocellulolytic enzymes per dry weight of protein in these samples are shown in Fig 4. The endoglucanase enzyme activities kept increasing from phase 1 to phase 5, whereas the cellobiohydrolase and beta-glucosidase activities both peaked at phase 4. The beta-xylosidase enzyme activity was highest at phase 2. Differences between the layers were significant for the results obtained for the above enzymes. Briefly, the activities in the upper layer were relatively higher than those in the other two layers. The activities of the lignin-modifying enzymes differed from those of the cellulases and hemicellulases. After oxidizing on ABTS and guaiacol, the highest laccase and peroxidase activities were observed in phase 1 and began to decrease in phase 2. From phase 3 to phase 5, both the laccase and peroxidase activities decreased significantly to a low level. The enzyme activities of these lignin-modifying enzymes did not differ significantly among the three layers.

### Lignin, cellulose and hemicellulose contents in the compost during G0119 growth

**Fig 5:**
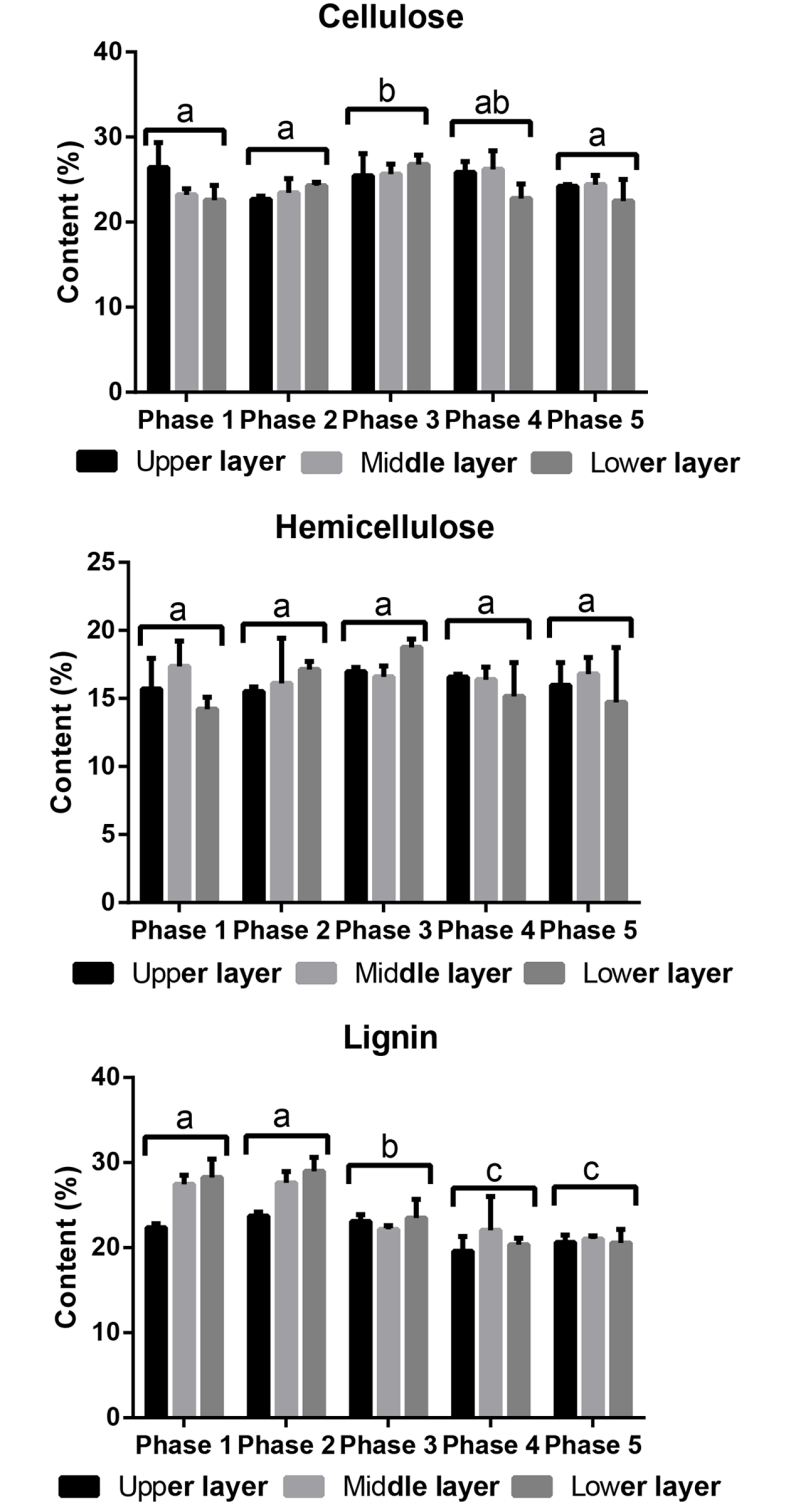
Lignocellulose compostion changes in the compost during the 5 *G*. *lucidum* G0119 growth phases.

The changes in the lignin, cellulose and hemicellulose contents in the compost are presented in Fig 5, and the monosaccharide composition of cellulose and hemicellulose are presented in Table 3. The lignin content in the samples was 23%-29% from phase 1 to phase 2. From phase 3 to phase 5, the contents decreased; the largest change of approximately 5.50% occurred from phase 2 to phase 3 in the middle and lower layers of the compost. The lignin content in the upper layer was relatively lower than the contents in the other two layers during growth. The hemicellulose in the sample was primarily composed of xylose, with low ratios of arabinose, galactose and mannose. However, the total hemicellulose content in the samples during growth did not change significantly at the level of 0.05. Cellulose was the main lignocellulose component in the compost and its content of it in the samples was approximately 24% at phase 1. The greatest cellulose content of 26.77% was detected in the lower layer of phase 3. From phase 3 to phase 5, the cellulose content decreased to a lower level of approximately 23%. The differences in both the cellulose and hemicellulose contents among the three compost layers were not significant at the level of 0.05. Generally, the cellulose and hemicellulose in the samples did not present great changes, suggesting that the growth of *G*. *lucidum* for one flush of fruiting bodies only degraded a small part of the lignocellulose in the substrate.

**Table 3:** Monosaccharide compositions of cellulose and hemicellulose in the composts during the 5 *G*. *lucidum* G0119 growth phases.

Samples (molar ratio %)		Ara	Gal	Glc	Xyl	Man
Phase 1	Upper layer	4.60±0.30	2.79±0.21	59.35±2.03	27.89±1.58	5.37±0.07
	Middle layer	6.03±0.44	3.05±0.23	53.65±3.12	32.13±2.41	5.15±0.32
	Lower layer	5.18±0.18	3.08±0.15	57.88±0.91	28.82±0.77	5.04±0.38
Phase 2	Upper layer	5.51±0.17	2.88±0.12	55.86±0.20	30.39±0.73	5.36±0.42
	Middle layer	7.35±0.72	2.70±0.33	55.99±3.86	28.57±4.18	5.39±0.07
	Lower layer	6.57±0.65	2.69±0.27	55.03±0.47	30.14±0.61	5.57±0.24
Phase 3	Upper layer	7.35±0.96	2.34±0.21	56.33±3.10	28.34±2.14	5.64±0.17
	Middle layer	6.81±0.22	2.17±0.12	57.15±1.64	28.41±1.38	5.45±0.08
	Lower layer	7.32±0.72	2.40±0.15	55.19±1.50	23.32±1.08	5.77±0.36
Phase 4	Upper layer	7.78±0.61	2.29±0.00	57.32±1.41	27.72±1.03	4.90±0.23
	Middle layer	7.44±0.64	2.23±0.00	58.19±0.16	26.99±0.23	5.14±0.26
	Lower layer	8.78±1.20	2.57±0.62	53.06±7.44	30.00±4.83	5.59±0.97
Phase 5	Upper layer	9.24±1.29	2.74±0.15	56.72±2.87	26.54±2.00	4.78±0.28
	Middle layer	9.75±0.51	2.56±0.03	55.62±0.80	27.09±0.64	4.98±0.12
	Lower layer	9.24±0.96	2.52±0.36	57.32±5.08	26.61±3.91	4.31±0.09

Note: Ara = arabinose, Gal = galactose, Glc = glucose, Xyl = xylose, Man = mannose

## Discussion and conclusion

In this study, we sequenced the genome of the *G*. *lucidum* strain G0119. Multiple strains of *Ganoderma* spp. have been sequenced at present. For instance, the genome size of strain CGMCC5.0026 (NCBI accession number: PRJNA71455) was reported to be 43.4 Mb in size, and containing 16,113 genes [11], of which 451 were CAZy genes. The reported genome size of strain *Ganoderma* sp. 10,597 SS1 in the JGI database is 39.52 Mb, and containing 12,910 genes [33]. Phylogenetic analysis of these two strains and *G*. *lucidum* G0119 indicated that strain G0119 was more closely related to strain CGMCC5.0026 than 10597 SS1, but that some gene loss has during its evolution [17]. Strain G0119 contained more CAZy protein genes than strain CGMCC5.0026, particularly with respect to the CE10 and CBM50 protein families, which primarily included esterases, chitinases and peptide enzymes. The number of CAZy genes identified in strain 10597 SS1 was much lower than that of strain G0119, possibly due to the use of the Sanger sequencing method instead of the next-generation sequencing approach used in this study [33]. Compared with the genomes of other wood-degrading fungal models, such as *Phanerochaete chrysosporium*, *Laccaria bicolor* and *Schizophyllum commune* [34–36], *G*. *lucidum* strain G0119 contained more GH and CE family genes, particularly those of the GH18, CE10 and CE16 families, which primarily consisted of chitinases, N-acetylglucosaminidases, arylesterases, and acetylesterases.

Previous transcriptome and proteome analyses of *G*. *lucidum* found that GH family coding genes were generally upregulated in the fruiting body phase compared to samples from the mycelial growth phase [12, 13]. Additionally, many genes involved in the fungal growth and fruiting body development in *G*. *lucidum* were identified. Certain mitogen-activated protein kinases, transcription factor and phosphoglucomutases were found that could regulate hyphal growth, primordium formation or biosynthesis of chitin and polysaccharides through carbohydrate metabolic pathways [37–39]. Our results revealed more information about gene expression and protein abundance changes of the lignocellulolytic enzymes during *G*. *lucidum* growth. Among these genes, the gene expression and protein abundance of lignin-modifying enzymes were observed primarily at high levels in phase 1 during mycelial growth. The changes in the enzyme activity of the lignin-modifying enzymes in *G*. *lucidum* G0119 also agreed with those results. However, genes encoding AA2 haem peroxidases were dominant, whereas laccases possessed the highest protein abundance in the secretomic results. In studies of other fungi, the expression of lignin-modifying enzymes was also observed to be significantly higher during mycelial growth in *A*. *bisporus* [32, 40]. Additionally, the expression of laccase-encoding genes was higher than that of peroxidases, which differed from that our observation in *G*. *lucidum* G0119. In *P*. *ostreatus*, *Lentinus edodes* and *L*. *tigrinus*, the laccase and peroxidase activities also found reached their maximum levels during the mycelial growth phase [41–43], suggesting that basidiomycetes primarily degraded lignin during the mycelial growth phase and that this degradation involved laccase and peroxidase. However, the lignin content decreased greatly after phase 3. This finding suggested that the degradation of lignin was not complete at phases 1 and 2 and that these lignin components were retained in the lignin precipitates. Among the lignin-modifying enzymes secreted by *G*. *lucidum* G0119, the most abundant was a laccase with a transmembrane domain, indicating that this type of lignin-degradation primarily occurred at the border of mycelial cells and the substrate. After breaking down the lignin structure and contacting the cellulose and hemicellulose surfaces, the degradation and utilization of cellulose and hemicellulose increased gradually as the fruiting bodies developed. The upregulated genes included 2 laccase-encoding genes that were upregulated in phase 2 when the primordium was formed. This finding is in agreement with the results from a study of *Volvaria volvacea*, in which the expression of one laccase gene was greatly increased during primordium growth, unlike the expression pattern of other laccase-encoding genes [44]. Studies of *P*. *ostreatus* and *Hypsizygus marmoreus* also observed that primordial growth was accelerated by increasing the expression of certain laccase genes [45, 46]. These findings suggest that certain genes in G0119 are also involved in primordium formation as well as in increasing lignin degradation. The upregulation of genes encoding AA2 haem peroxidases, endoglucanases, xylanases, endogalactosidases and β-galactosidases during phase 1 indicated that the lignin and hemicellulose-degrading abilities of G0119 both increased during mycelial growth. High expression of hemicellulase and lignin-modifying enzyme-encoding genes was observed during this same phase, possibly because lignin and hemicellulose were more closely distributed with each other than with cellulose [47].

From phase 3 to phase 5, several endoglucanase, xylanase and glucosidase-encoding genes were upregulated, which was also in agreement with the changes in enzyme activity. These data agreed with the transcriptomic and proteomic results obtained from *A*. *bisporus* [32] and the enzyme activity results from *P*. *ostreatus*, *Lentinus edodes* and *L*. *tigrinus* [41–43], indicating that cellulase and hemicellulase gene expression was potentially correlated with fruiting body formation. The cellulose content in the compost was also decreased during this period. CBH was the most abundant secreted enzyme during these phases, indicating that the hydrolysis of cellulose or cellotetraose to cellobiose was a dominant process that was involved in substrate degradation throughout the long growth cycle. In *A*. *bisporus*, the expression of cellulose- and hemicellulase-encoding genes was also observed to be upregulated during the first flush fruiting phase. The most abundant extracellular proteins in *A*. *bisporus* were CBH isozymes [32]. The difference between these two basidiomycetes was that the most abundant CBH isozymes secreted by G0119 were all type I CBHs from the GH6 family, whereas the most abundant CBH in *A*. *bisporus* was a type II enzyme from the GH7 family. This observation indicated that these isozymes are selectively produced in different species. The dominant isozymes contributing to substrate degradation may represent targets for the regulation of lignocellulose degradation to accelerate the growth of *G*. *lucidum*.

Furthermore, the brief increase in the expression of the entire genome, including the CAZy genes at phase 5, was greater than that observed during the other growth phases. Among them, 21 lignocellulolytic enzymes-encoding genes were also upregulated during this phase, and a large number of basidiospores was produced. As one physiological activity of reproduction, spore formation may carry a high carbohydrate and nutritional metabolic cost, resulting in high cellulase and hemicellulase expression and secretion and thus a carbohydrate metabolic peak during phase 5. Some of the spore composition was found to derive from carbohydrates in the substrate mycelial, which was suggested in reports of basidiomycetes, such as *Coprinus cinereus* and *Flammulina velutipes* [48, 49]. Although data for this growth phase are still lacking, it can be hypothesized that carbohydrate metabolism potentially plays an important role in basidiospore production in *G*. *lucidum*.

## Data deposition

The sequencing data (NCBI: PRJNA406843), the *G*. *lucidum* G0119 genome sequence (NCBI: SRR6026942) and the raw Illumina sequencing data during the 5 growth phases, performed in duplicates (NCBI: SRR6026942, SRR6027258, SRR6027261, SRR6027352, SRR6037451, SRR6037587, SRR6037726, SRR6040102, SRR6040213 and SRR6043945), were deposited at NCBI.

## Supporting information

S1 Fig Heatmap of CAZy protein expression during the 5 *G*. *lucidum* G0119 growth phases. The gene expression levels during phase 3 and 4 were generally similar. Based on the expression levels, these genes were divided into four groups. Group 3 showed the highest expression levels and contained 87 genes (Left part), 10 of which were lignocellulolytic enzymes.
(PDF)

S2 Fig Heatmap of CAZy protein abundance in secreted protein samples during the 5 *G*. *lucidum* G0119 growth phases. The protein abundances between phases 3 and 4 and between phases 1 and 2 were similar. The most abundant CAZy proteins were CBH, endopolygalacturonase, α-galactosidase, xyloglucanase and endoxylanase.
(PDF)

S3 Fig Comparison of the expression and protein abundance of lignocellulolytic enzymes during the 5 growth phases of *G*. *lucidum* G0119. Lignocellulolytic enzymes were classified into 9 groups based on their function and compared. Ninety-five and 61 enzymes were detected in the transcriptomic and secretomic analyses, respectively. The brief expression of endoglucanase, CBH and mannosidase increased from phase 1 to phase 5, and their overall protein abundance also changed accordingly. One to two isozymes in each group had a higher protein abundance than the other enzymes.
(PDF)

S4 Fig Protein concentrations of extracellular enzyme samples during growth of *G*. *lucidum* G0119. (TIF)

S1 Table Distribution of CAZy family proteins in *G*. *lucidum* G0119. (DOCX)

S2 Table Putative lignocellulolytic enzymes in *G*. *lucidum* G0119. (DOCX)

S3 Table Highly expressed CAZy protein-encoding genes in *G*. *lucidum* G0119 and their functions. (DOCX)

S4 Table Key words for the identification of the lignocellulolytic genes by annotation. (PDF)

## Abbreviations

ABF: alpha-N-arabinofuranosidase
ABN: endo-1,5-alpha-L-arabinosidase
BGA: beta-galactosidase
BGL: beta-glucosidase
BXL: exo-1,4-beta-xylosidase
CAZy: Carbohydrate Active enZymes
CBH: cellobiohydrolase
EG: endoglucanase/endo-beta-1,4-glucanase
EGA: endo-1,4-beta-galactosidase
EXG: exoglucanase
MAN: alpha-mannosidase
MANB: beta-mannosidase
MnP: manganese peroxidase
VP: versatile peroxidase
XEG: xyloglucan-specific endo-beta-1,4-glucanase
XYL: endo-1,4-beta-xylanase
