# Supplementary figures and images for: Investigation of lignocellulolytic enzymes during different growth phases of Ganoderma lucidum strain G0119 using genomic, transcriptomic and secretomic analyses
Source: PLoS One. 2018 May 31;13(5):e0198404. doi: 10.1371/journal.pone.0198404 (PMC5979026; doi:10.1371/journal.pone.0198404)

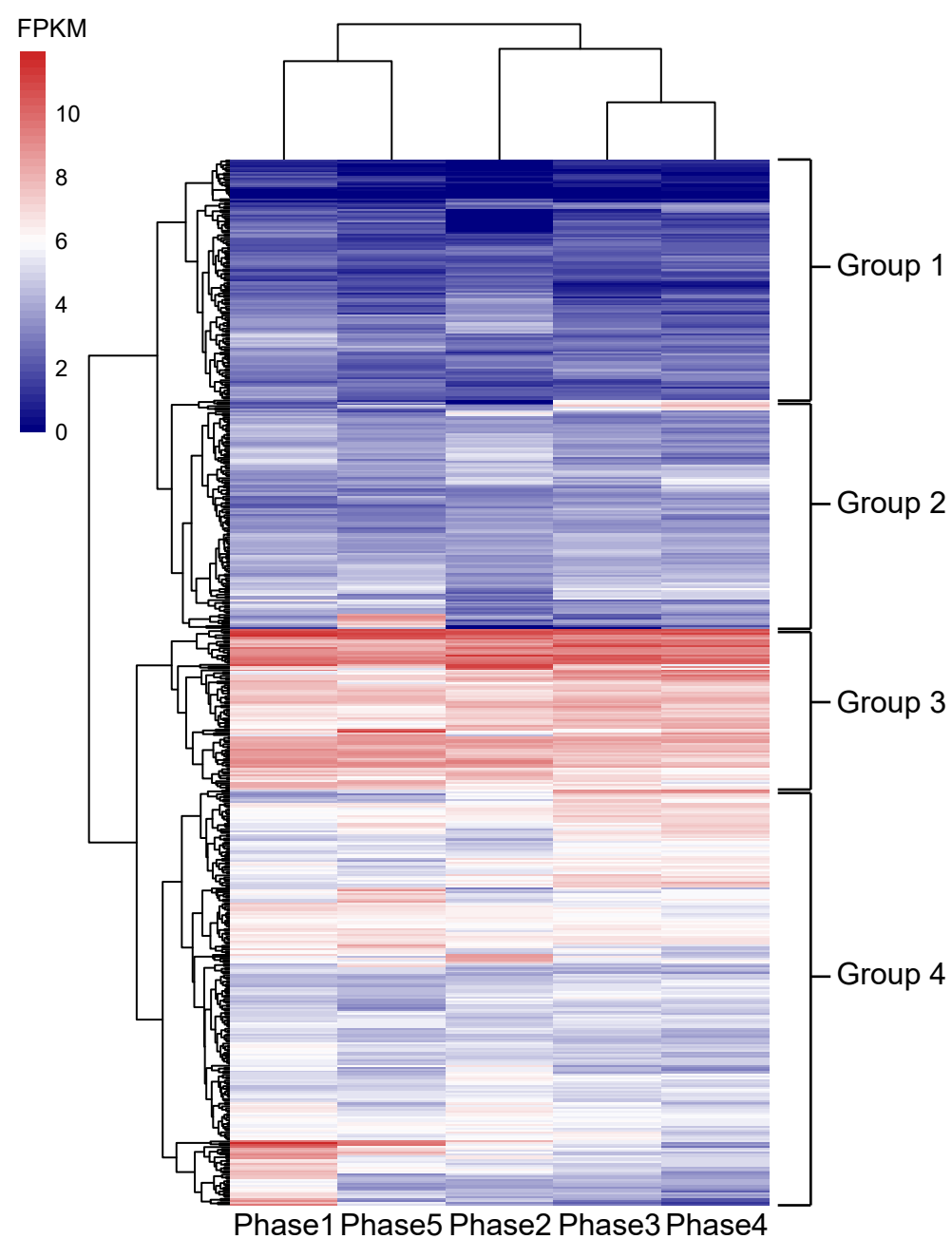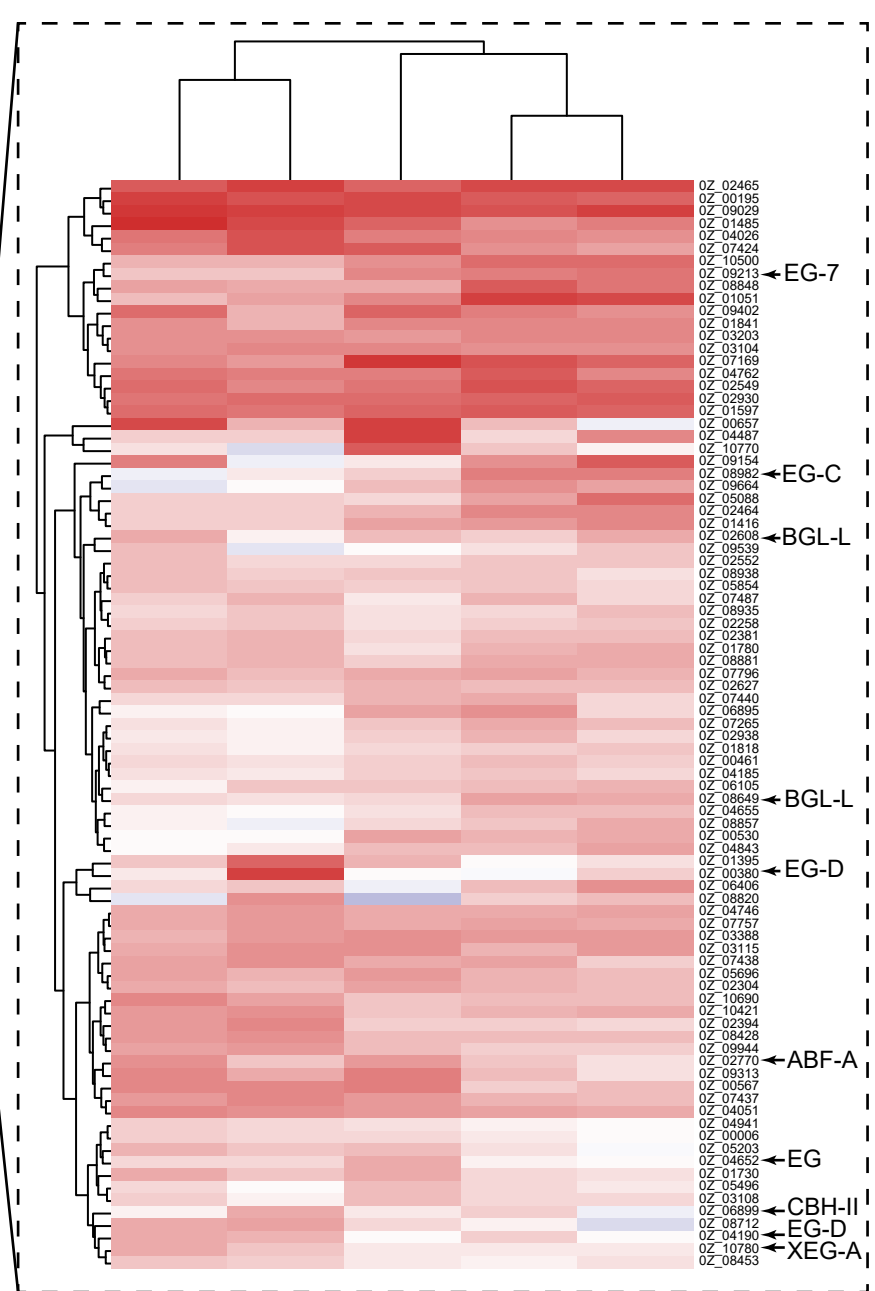

Supplement: S1 Fig — The gene expression levels during phase 3 and 4 were generally similar. Based on the expression levels, these genes were divided into four groups. Group 3 showed the highest expression levels and contained 87 genes (Left part), 10 of which were lignocellulolytic enzymes. (PDF) [file pone.0198404.s001.pdf]

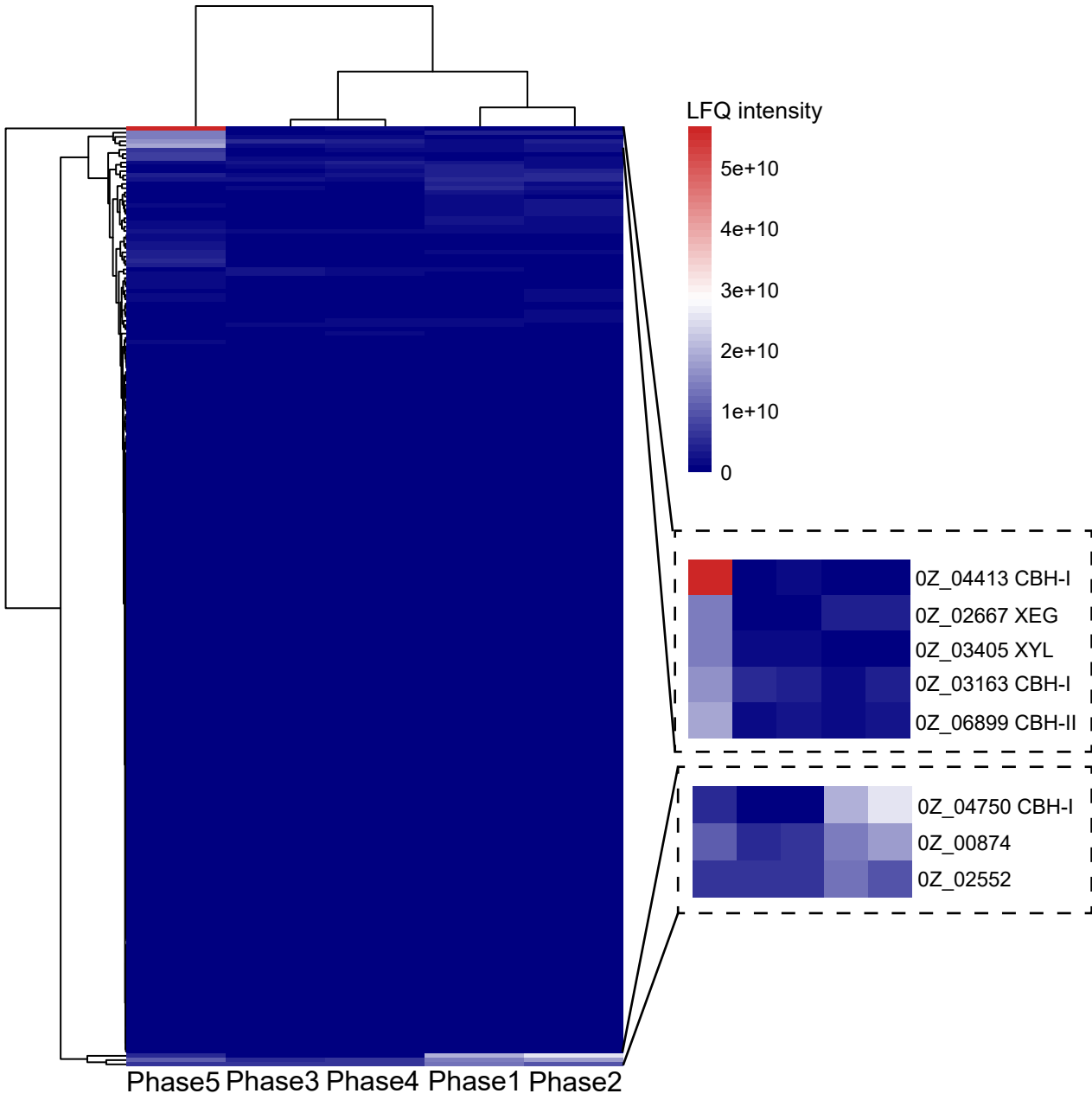

Supplement: S2 Fig — The protein abundances between phases 3 and 4 and between phases 1 and 2 were similar. The most abundant CAZy proteins were CBH, endopolygalacturonase, α-galactosidase, xyloglucanase and endoxylanase. (PDF) [file pone.0198404.s002.pdf]

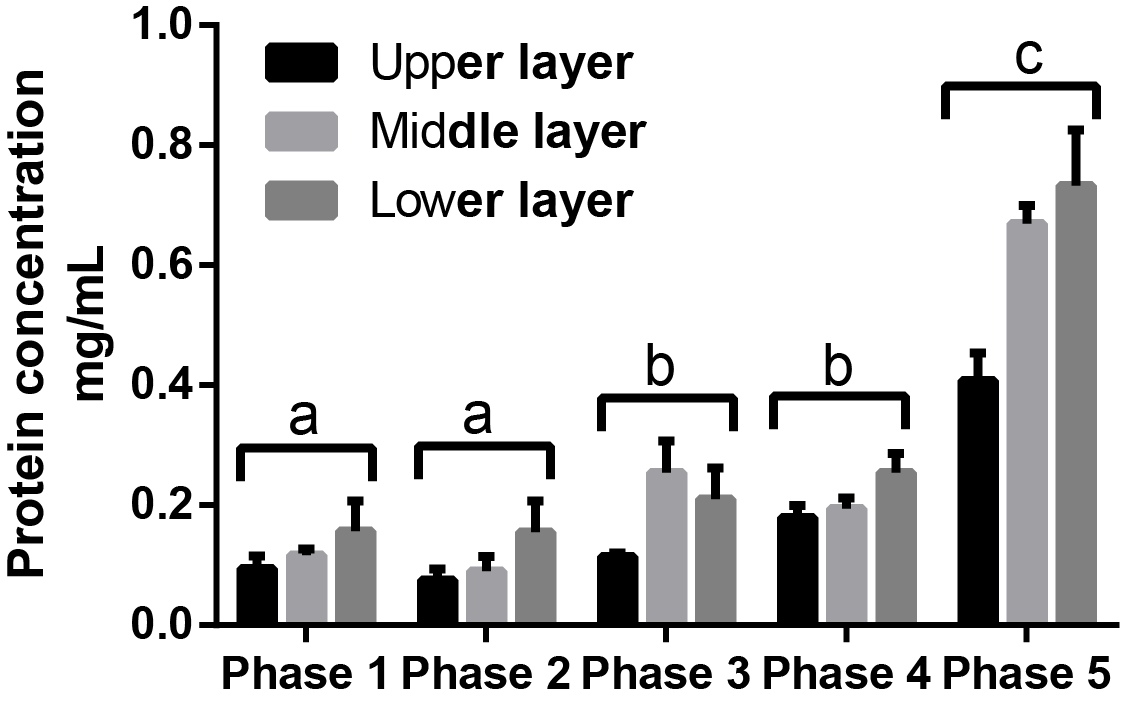

Supplement: S4 Fig — (TIF) [file pone.0198404.s004.tif]
