# Supplementary material for: Investigation of lignocellulolytic enzymes during different growth phases of Ganoderma lucidum strain G0119 using genomic, transcriptomic and secretomic analyses
Source: PLoS One. 2018 May 31;13(5):e0198404. doi: 10.1371/journal.pone.0198404 (PMC5979026; doi:10.1371/journal.pone.0198404)
Supplement: S3 Fig — Lignocellulolytic enzymes were classified into 9 groups based on their function and compared. Ninety-five and 61 enzymes were detected in the transcriptomic and secretomic analyses, respectively. The brief expression of endoglucanase, CBH and mannosidase increased from phase 1 to phase 5, and their overall protein abundance also changed accordingly. One to two isozymes in each group had a higher protein abundance than the other enzymes. (PDF) [file pone.0198404.s003.pdf]

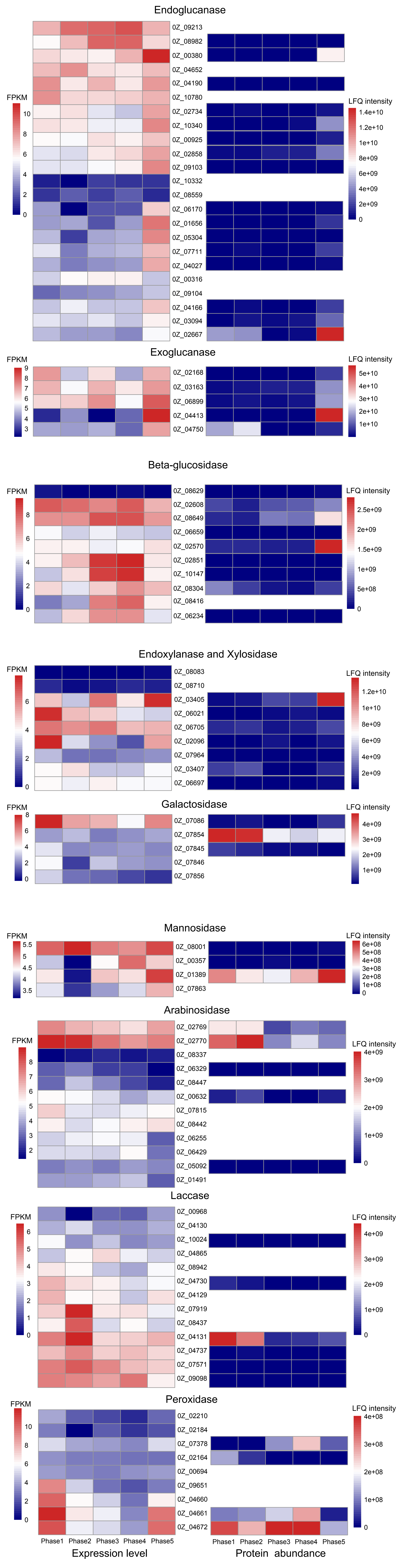

Endoglucanase
FPKM
10
8
6
4
2
0
0Z_09213
0Z_08982
0Z_00380
0Z_04652
0Z_04190
0Z_10780
0Z_02734
0Z_10340
0Z_00925
0Z_02858
0Z_09103
0Z_10332
0Z_08559
0Z_06170
0Z_01656
0Z_05304
0Z_07711
0Z_04027
0Z_00316
0Z_09104
0Z_04166
0Z_03094
0Z_02667
LFQ intensity
1.4e+10
1.2e+10
1e+10
8e+09
6e+09
4e+09
2e+09
0
Exoglucanase
FPKM
9
8
7
6
5
4
3
0Z_02168
0Z_03163
0Z_06899
0Z_04413
0Z_04750
LFQ intensity
5e+10
4e+10
3e+10
2e+10
1e+10
Beta-glucosidase
FPKM
8
6
4
2
0
0Z_08629
0Z_02608
0Z_08649
0Z_06659
0Z_02570
0Z_02851
0Z_10147
0Z_08304
0Z_08416
0Z_06234
LFQ intensity
2.5e+09
2e+09
1.5e+09
1e+09
5e+08
0
Endoxylanase and Xylosidase
FPKM
6
4
2
0
0Z_08083
0Z_08710
0Z_03405
0Z_06021
0Z_06705
0Z_02096
0Z_07964
0Z_03407
0Z_06697
LFQ intensity
1.2e+10
1e+10
8e+09
6e+09
4e+09
2e+09
Galactosidase
FPKM
8
6
4
2
0
0Z_07086
0Z_07854
0Z_07845
0Z_07846
0Z_07856
LFQ intensity
4e+09
3e+09
2e+09
1e+09
Mannosidase
FPKM
5.5
5
4.5
4
3.5
0Z_08001
0Z_00357
0Z_01389
0Z_07863
LFQ intensity
6e+08
5e+08
4e+08
3e+08
2e+08
1e+08
0
Arabinosidase
FPKM
8
7
6
5
4
3
2
0Z_02769
0Z_02770
0Z_08337
0Z_06329
0Z_08447
0Z_00632
0Z_07815
0Z_08442
0Z_06255
0Z_06429
0Z_05092
0Z_01491
LFQ intensity
4e+09
3e+09
2e+09
1e+09
0
Laccase
FPKM
6
5
4
3
2
1
0
0Z_00968
0Z_04130
0Z_10024
0Z_04865
0Z_08942
0Z_04730
0Z_04129
0Z_07919
0Z_08437
0Z_04131
0Z_04737
0Z_07571
0Z_09098
LFQ intensity
4e+09
3e+09
2e+09
1e+09
0
Peroxidase
FPKM
10
8
6
4
2
0
0Z_02210
0Z_02184
0Z_07378
0Z_02164
0Z_00694
0Z_09651
0Z_04660
0Z_04661
0Z_04672
LFQ intensity
4e+08
3e+08
2e+08
1e+08
0
Phase1
Phase2
Phase3
Phase4
Phase5
Expression level
Phase1
Phase2
Phase3
Phase4
Phase5
Protein abundance
