# Supplementary material for: Investigation of lignocellulolytic enzymes during different growth phases of Ganoderma lucidum strain G0119 using genomic, transcriptomic and secretomic analyses
Source: PLoS One. 2018 May 31;13(5):e0198404. doi: 10.1371/journal.pone.0198404 (PMC5979026; doi:10.1371/journal.pone.0198404)
Supplement: S1 Table — (DOCX) [file pone.0198404.s005.docx]

Table S1. Distribution of CAZy family protein in *G. lucidum* G0119

| Family No. | Gene No. | Family No. | Gene No. | Family No. | Gene No. | Family No. | Gene No. | Family No. | Gene No. | Family No. | Gene No. |  |  | |
| --- | --- | --- | --- | --- | --- | --- | --- | --- | --- | --- | --- | --- | --- | --- |
| GH1 | 3 | GH28 | 9 | GH78 | 4 | GH15 | 3 | GH51 | 2 | GH109 | 6 |  |  | |
| GH2 | 3 | GH30 | 2 | GH79 | 11 | GH16 | 32 | GH53 | 1 | GH115 | 3 |  |  | |
| GH3 | 12 | GH31 | 8 | GH85 | 1 | GH17 | 1 | GH55 | 3 | GH125 | 1 |  |  | |
| GH5 | 18 | GH32 | 1 | GH88 | 1 | GH18 | 34 | GH63 | 1 | GH128 | 6 |  |  | |
| GH6 | 2 | GH35 | 5 | GH89 | 2 | GH20 | 5 | GH71 | 6 | GH131 | 3 |  |  | |
| GH7 | 3 | GH37 | 2 | GH92 | 5 | GH23 | 1 | GH72 | 1 | GH133 | 1 |  |  | |
| GH9 | 1 | GH38 | 1 | GH93 | 1 | GH25 | 2 | GH74 | 2 | GH135 | 1 |  |  | |
| GH10 | 6 | GH43 | 12 | GH95 | 2 | GH27 | 5 | GH76 | 2 |  |  |  |  | |
| GH12 | 3 | GH45 | 1 | GH99 | 1 |  |  |  |  |  |  |  |  | |
| GH13 | 6 | GH47 | 9 | GH105 | 5 |  |  |  |  | Total GH | 262 |  |  | |
| Family No. | Gene No. | Family No. | Gene No. | Family No. | Gene No. | Family No. | Gene No. | Family No. | Gene No. | Family No. | Gene No. | Family No. | | Gene No. |
| GT1 | 6 | GT22 | 4 | GT50 | 11 | CE1 | 15 | AA1 | 2 | CBM1 | 1 | PL8 | 3 | |
| GT2 | 12 | GT24 | 1 | GT57 | 1 | CE2 | 1 | AA2 | 10 | CBM5 | 1 | PL12 | 1 | |
| GT3 | 1 | GT28 | 2 | GT58 | 1 | CE4 | 6 | AA3 | 32 | CBM13 | 13 | PL14 | 6 | |
| GT4 | 4 | GT31 | 1 | GT59 | 1 | CE8 | 3 | AA4 | 2 | CBM19 | 3 | PL15 | 1 | |
| GT5 | 1 | GT32 | 4 | GT65 | 2 | CE9 | 1 | AA5 | 9 | CBM20 | 1 |  |  | |
| GT8 | 6 | GT33 | 1 | GT66 | 1 | CE10 | 44 | AA6 | 2 | CBM21 | 2 |  |  | |
| GT15 | 3 | GT35 | 1 | GT68 | 1 | CE12 | 2 | AA7 | 12 | CBM32 | 1 |  |  | |
| GT17 | 1 | GT39 | 3 | GT69 | 3 | CE14 | 1 | AA8 | 1 | CBM48 | 1 |  |  | |
| GT20 | 3 | GT48 | 2 | GT76 | 2 | CE15 | 2 | AA9 | 15 | CBM50 | 10 |  |  | |
| GT21 | 1 | GT49 | 1 | GT90 | 1 | CE16 | 16 |  |  |  |  |  |  | |
|  |  |  |  |  |  |  |  |  |  |  |  |  |  | |
|  |  |  |  | Total GT | 72 | Total CE | 91 | Total AA | 85 | Total CBM | 33 | Total PL | 11 | |
