# Supplementary material for: Investigation of lignocellulolytic enzymes during different growth phases of Ganoderma lucidum strain G0119 using genomic, transcriptomic and secretomic analyses
Source: PLoS One. 2018 May 31;13(5):e0198404. doi: 10.1371/journal.pone.0198404 (PMC5979026; doi:10.1371/journal.pone.0198404)
Supplement: S2 Table — (DOCX) [file pone.0198404.s006.docx]

Table S2. The putative lignocellulolytic enzymes in *G. lucidum* G0119

| Category | GeneID | CAZy family | Function (enzyme abbreviations) | Amino Acids | Isoelectric point | SignalP | TMHMM |
| --- | --- | --- | --- | --- | --- | --- | --- |
| Cellulase | 0Z_02858 | GH5 | Endoglucanase EGII (EG-II) | 415 | 4.17 | 1-19 | — |
|  | 0Z_03094 | GH5 | Endoglucanase EGII (EG-II) | 372 | 4.44 | 1-16 | — |
|  | 0Z_07711 | GH5 | Endoglucanase 3 (EG-3) | 390 | 4.27 | 1-18 | — |
|  | 0Z_08982 | GH5 | Endoglucanase C (EG-C) | 475 | 6.17 | — | — |
|  | 0Z_00316 | GH9 | Endoglucanase A (EG-A) | 580 | 5.14 | 1-19 | 542-564 |
|  | 0Z_02734 | GH12 | Xyloglucan-specific endo-beta-1,4-glucanase A (XEG-A) | 257 | 4.46 | 1-19 | — |
|  | 0Z_10340 | GH12 | Xyloglucan-specific endo-beta-1,4-glucanase A (XEG-A) | 242 | 4.14 | 1-18 | — |
|  | 0Z_10780 | GH12 | Xyloglucan-specific endo-beta-1,4-glucanase A (XEG-A) | 287 | 6.82 | — | — |
|  | 0Z_06170 | GH45 | Endoglucanase type K (EG-K) | 230 | 4.55 | 1-22 | — |
|  | 0Z_02667 | GH74 | Xyloglucanase (XEG) | 839 | 4.66 | 1-21 | — |
|  | 0Z_04166 | GH131 | Endoglucanase-4 (EG-4) | 328 | 4.53 | 1-17 | — |
|  | 0Z_00925 | CBM1 | Endoglucanase type F (EG-F) | 245 | 4.10 | 1-20 | — |
|  | 0Z_00380 | AA9 | Endo-beta-1,4-glucanase D (EG-D) | 228 | 5.03 | 1-16 | — |
|  | 0Z_01656 | AA9 | Endo-beta-1,4-glucanase D (EG-D) | 267 | 4.19 | 1-17 | — |
|  | 0Z_04027 | AA9 | Endo-beta-1,4-glucanase D (EG-D) | 224 | 5.11 | 1-17 | — |
|  | 0Z_04190 | AA9 | Endo-beta-1,4-glucanase D / endoglucanase II (EG-D/EG-II) | 203 | 5.00 | 1-20 | — |
|  | 0Z_04652 | AA9 | Beta-1,4-endoglucanase (EG) | 337 | 8.52 | 1-22 | — |
|  | 0Z_05304 | AA9 | Endo-beta-1,4-glucanase D (EG-D) | 242 | 4.39 | 1-18 | — |
|  | 0Z_08559 | AA9 | Endo-beta-1,4-glucanase D (EG-D) | 408 | 5.88 | — | — |
|  | 0Z_09103 | AA9 | Endo-beta-1,4-glucanase D (EG-D) | 256 | 4.44 | 1-19 | — |
|  | 0Z_09104 | AA9 | Endo-beta-1,4-glucanase D (EG-D) | 256 | 4.83 | 1-19 | — |
|  | 0Z_09213 | AA9 | Endoglucanase-7 (EG-7) | 333 | 6.12 | 1-19 | — |
|  | 0Z_10332 | AA9 | Endoglucanase-7 / endoglucanase-4 (EG-7/EG-4) | 261 | 4.58 | 1-20 | — |
|  | 0Z_06676 | GH6 | Exoglucanase 3 / cellobiohydrolaseII (EXG-3/CBH-II) | 178 | 4.73 | — | — |
|  | 0Z_06899 | GH6 | Exoglucanase 3 / cellobiohydrolaseII (EXG-3/CBH-II) | 449 | 4.28 | 1-20 | — |
|  | 0Z_03163 | GH7 | Exoglucanase 1 / cellobiohydrolaseI (EXG-1/CBH-I) | 456 | 4.33 | 1-18 | — |
|  | 0Z_04413 | GH7 | Exoglucanase 1 / cellobiohydrolaseI (EXG-1/CBH-I) | 458 | 4.15 | 1-18 | — |
|  | 0Z_04750 | GH7 | Exoglucanase 1 / cellobiohydrolaseI (EXG-1/CBH-I) | 457 | 4.44 | 1-18 | — |
|  | 0Z_02168 | CE15 | Exoglucanase 3 (EXG-3) | 467 | 4.70 | 1-21 | — |
|  | 0Z_02851 | GH1 | Beta-glucosidase 1A (BGL-1A) | 443 | 5.31 | — | — |
|  | 0Z_06659 | GH1 | Beta-glucosidase 24 (BGL-24) | 521 | 5.56 | 1-25 | — |
|  | 0Z_08416 | GH1 | Beta-glucosidase 1B (BGL-1B) | 529 | 5.12 | — | — |
|  | 0Z_02570 | GH3 | Beta-glucosidase L (BGL-L) | 1465 | 5.33 | — | — |
|  | 0Z_02608 | GH3 | Beta-glucosidase L (BGL-L) | 798 | 5.22 | — | — |
|  | 0Z_06234 | GH3 | Beta-glucosidase I (BGL) | 799 | 5.78 | — | — |
|  | 0Z_08304 | GH3 | Periplasmic beta-glucosidase (P-BGL) | 819 | 4.84 | 1-31 | — |
|  | 0Z_08629 | GH3 | Beta-glucosidase L (BGL-L) | 679 | 5.16 | 1-24 | — |
|  | 0Z_08649 | GH3 | Beta-glucosidase L (BGL-L) | 786 | 4.85 | — | — |
|  | 0Z_10147 | GH3 | Beta-glucosidase I (BGL-L) | 861 | 5.72 | — | — |
| Hemicellulase | 0Z_02096 | GH10 | Endo-1,4-beta-xylanase (XYL) | 338 | 4.87 | 1-18 | — |
|  | 0Z_03405 | GH10 | Endo-1,4-beta-xylanase (XYL) | 419 | 5.49 | 1-19 | — |
|  | 0Z_03407 | GH10 | Endo-1,4-beta-xylanase (XYL) | 381 | 4.51 | 1-20 | — |
|  | 0Z_06021 | GH10 | Endo-1,4-beta-xylanase (XYL) | 353 | 5.21 | 1-20 | — |
|  | 0Z_08083 | GH10 | Endo-1,4-beta-xylanase (XYL) | 360 | 5.17 | 1-17 | — |
|  | 0Z_06697 | GH3 | exo-1,4-beta-xylosidase (BXL) | 777 | 4.99 | 1-24 | — |
|  | 0Z_06705 | GH3 | exo-1,4-beta-xylosidase (BXL) | 774 | 4.85 | 1-24 | — |
|  | 0Z_07964 | GH3 | exo-1,4-beta-xylosidase (BXL) | 787 | 4.83 | 1-28 | — |
|  | 0Z_08710 | GH3 | exo-1,4-beta-xylosidase (BXL) | 495 | 5.67 | — | — |
|  | 0Z_07086 | GH53 | arabinogalactan endo-1,4-beta-galactosidase A (EGA-A) | 343 | 4.78 | 1-18 | — |
|  | 0Z_07845 | GH35 | beta-galactosidase C (BGA-C) | 2130 | 4.84 | — | — |
|  | 0Z_07846 | GH35 | beta-galactosidase B (BGA-B) | 2811 | 4.77 | 1-22 | — |
|  | 0Z_07854 | GH35 | beta-galactosidase A (BGA-A) | 6522 | 4.59 | 1-15 | — |
|  | 0Z_07856 | GH35 | beta-galactosidase B (BGA-B) | 2772 | 4.78 | — | — |
|  | 0Z_07859 | GH35 | beta-galactosidase A (BGA-A) | 2382 | 4.82 | — | — |
|  | 0Z_08001 | GH38 | Alpha-mannosidase (MAN) | 1116 | 6.54 | — | — |
|  | 0Z_00357 | GH2 | beta-mannosidase B (MANB-B) | 866 | 5.73 | — | — |
|  | 0Z_01389 | GH2 | beta-mannosidase A (MANB-A) | 951 | 4.92 | 1-20 | — |
|  | 0Z_07863 | GH2 | beta-mannosidase B (MANB-B) | 901 | 5.30 | — | — |
|  | 0Z_00632 | GH43 | arabinan endo-1,5-alpha-L-arabinosidase A (ABN-A) | 640 | 5.49 | — | — |
|  | 0Z_05092 | GH43 | arabinan endo-1,5-alpha-L-arabinosidase A (ABN-A) | 342 | 4.03 | 1-19 | — |
|  | 0Z_06255 | GH43 | arabinan endo-1,5-alpha-L-arabinosidase A (ABN-A) | 329 | 6.29 | 1-17 | — |
|  | 0Z_06329 | GH43 | arabinan endo-1,5-alpha-L-arabinosidase A (ABN-A) | 332 | 8.61 | 1-19 | — |
|  | 0Z_06429 | GH43 | arabinan endo-1,5-alpha-L-arabinosidase A (ABN-A) | 327 | 5.93 | 1-19 | — |
|  | 0Z_07815 | GH43 | Arabinan endo-1,5-alpha-L-arabinosidase (ABN) | 319 | 9.05 | 1-20 | — |
|  | 0Z_08337 | GH43 | arabinan endo-1,5-alpha-L-arabinosidase A (ABN-A) | 328 | 4.41 | 1-19 | — |
|  | 0Z_08442 | GH43 | Arabinan endo-1,5-alpha-L-arabinosidase (ABN) | 336 | 6.99 | 1-20 | — |
|  | 0Z_08447 | GH43 | Arabinan endo-1,5-alpha-L-arabinosidase (ABN) | 318 | 5.09 | 1-19 | — |
|  | 0Z_01491 | GH51 | alpha-N-arabinofuranosidase C (ABF-C) | 1599 | 4.95 | 1-25 | — |
|  | 0Z_02769 | GH51 | alpha-N-arabinofuranosidase A (ABF-A) | 732 | 5.07 | — | — |
|  | 0Z_02770 | GH51 | alpha-N-arabinofuranosidase A (ABF-A) | 783 | 5.04 | 1-17 | — |
| Lignin-modifying enzyme | 0Z_00968 | AA1 | Laccase-4 (LACC-4) | 519 | 4.87 | 1-22 | 7-29 |
|  | 0Z_04129 | AA1 | Laccase (LACC) | 614 | 6.38 | 1-24 | — |
|  | 0Z_04130 | AA1 | Laccase (LACC) | 526 | 5.18 | 1-22 | — |
|  | 0Z_04131 | AA1 | Laccase (LACC) | 520 | 4.66 | 1-21 | 7-24 |
|  | 0Z_04730 | AA1 | Laccase (LACC) | 520 | 5.58 | 1-21 | — |
|  | 0Z_04737 | AA1 | Laccase-2 (LACC-2) | 521 | 4.78 | 1-21 | — |
|  | 0Z_04865 | AA1 | Laccase-4 (LACC-4) | 541 | 4.50 | 1-23 | — |
|  | 0Z_07571 | AA1 | Laccase (LACC) | 521 | 5.14 | 1-21 | 7-29 |
|  | 0Z_07919 | AA1 | Laccase-4 (LACC-4) | 519 | 5.08 | 1-20 | 7-29 |
|  | 0Z_08437 | AA1 | Laccase-4 (LACC-4) | 523 | 4.94 | 1-24 | — |
|  | 0Z_08942 | AA1 | Laccase-2 (LACC-2) | 498 | 5.26 | 1-21 | 7-29 |
|  | 0Z_09098 | AA1 | Laccase-4 (LACC-4) | 532 | 4.54 | 1-34 | — |
|  | 0Z_10024 | AA1 | Laccase-2 (LACC-2) | 517 | 5.52 | 1-17 | 5-27 |
|  | 0Z_02164 | AA2 | Versatile peroxidase / manganese peroxidase (VP/MNP) | 366 | 4.29 | 1-18 | — |
|  | 0Z_02210 | AA2 | Manganese peroxidase 3 (MNP-3) | 365 | 4.21 | 1-21 | — |
|  | 0Z_04660 | AA2 | Manganese peroxidase 3 (MNP-3) | 362 | 4.36 | 1-19 | — |
|  | 0Z_04661 | AA2 | Ligninase C / manganese peroxidase (MNP) | 364 | 4.27 | 1-21 | — |
|  | 0Z_04672 | AA2 | Ligninase C / manganese peroxidase (MNP) | 366 | 4.29 | 1-18 | — |
|  | 0Z_02184 | AA2 | Versatile peroxidase VPL2 / manganese peroxidase (VP-L2/MNP) | 197 | 5.16 | 1-18 | — |
|  | 0Z_00694 | AA2 | Versatile peroxidase VPL1 (VP-L1) | 355 | 4.97 | 1-18 | — |
|  | 0Z_07378 | AA2 | Versatile peroxidase VPL2 (VP-L2) | 361 | 4.36 | 1-18 | — |
|  | 0Z_09651 | AA2 | Versatile peroxidase VPL1 / manganese-repressed peroxidase (VP-L1) | 361 | 4.67 | 1-18 | — |
