# Supplementary material for: Investigation of lignocellulolytic enzymes during different growth phases of Ganoderma lucidum strain G0119 using genomic, transcriptomic and secretomic analyses
Source: PLoS One. 2018 May 31;13(5):e0198404. doi: 10.1371/journal.pone.0198404 (PMC5979026; doi:10.1371/journal.pone.0198404)
Supplement: S3 Table — (DOCX) [file pone.0198404.s007.docx]

Table S3. Highly expressed genes of CAZy protein in *G. lucidum* G0119 and their functions

| GeneID | CAZy family | Function |
| --- | --- | --- |
| 0Z_08428 | GH10 | Inosine-5'-monophosphate dehydrogenase |
| 0Z_10780 | GH12 | Probable xyloglucan-specific endo-beta-1,4-glucanase |
| 0Z_00195 | GH128 | β-1,3-glucanase (EC 3.2.1.39) |
| 0Z_10500 | GH128 | β-1,3-glucanase (EC 3.2.1.40) |
| 0Z_05496 | GH15 | Glucoamylase |
| 0Z_01597 | GH16 | Probable glycosidase |
| 0Z_02381 | GH16 | Probable glycosidase |
| 0Z_02394 | GH16 | Probable glycosidase |
| 0Z_03203 | GH16 | Beta-glucan synthesis-associated protein |
| 0Z_04026 | GH16 | Probable glycosidase |
| 0Z_07437 | GH16 | Beta-1,3-glucan-binding protein |
| 0Z_07438 | GH16 | Beta-1,3-glucan-binding protein |
| 0Z_07487 | GH16 | Probable glycosidase |
| 0Z_08848 | GH16 | Probable glycosidase |
| 0Z_08857 | GH16 | Probable endo-1,3(4)-beta-glucanase |
| 0Z_03388 | GH17 | Probable glucan endo-1,3-beta-glucosidase |
| 0Z_02930 | GH18 | Chitinase |
| 0Z_04655 | GH18 | Endochitinase |
| 0Z_02552 | GH27 | Alpha-galactosidase |
| 0Z_02608 | GH3 | Probable beta-glucosidase |
| 0Z_08649 | GH3 | Probable beta-glucosidase |
| 0Z_07265 | GH37 | Trehalase |
| 0Z_05088 | GH47 | Mannosyl-oligosaccharide 1,2-alpha-mannosidase |
| 0Z_08453 | GH47 | Mannosyl-oligosaccharide 1,2-alpha-mannosidase |
| 0Z_01780 | GH5 | Probable glucan 1,3-beta-glucosidase |
| 0Z_04762 | GH5 | Glucan 1,3-beta-glucosidase |
| 0Z_06105 | GH5 | Probable glucan 1,3-beta-glucosidase |
| 0Z_08982 | GH5 | Endoglucanase |
| 0Z_02770 | GH51 | Probable alpha-N-arabinofuranosidase |
| 0Z_06899 | GH6 | Exocellobiohydrolase |
| 0Z_02464 | GH72 | 1,3-beta-glucanosyltransferase |
| 0Z_08881 | GH79 | Phosphoglycan α-1,2-D-arabinopyranosyltransferase 1 (EC 2.4.2.-) |
| 0Z_01841 | GT15 | Probable mannosyltransferase |
| 0Z_02258 | GT2 | Chitin synthase |
| 0Z_04746 | GT2 | Chitin synthase |
| 0Z_07440 | GT2 | cellulose synthase or chitin synthase |
| 0Z_09944 | GT2 | Dolichol-phosphate mannosyltransferase |
| 0Z_10421 | GT2 | Polysaccharide synthase |
| 0Z_10690 | GT2 | Dolichyl-phosphate beta-glucosyltransferase |
| 0Z_03108 | GT21 | Ceramide glucosyltransferase |
| 0Z_08935 | GT3 | Glycogen [starch] synthase |
| 0Z_00461 | GT32 | Mannosyl phosphorylinositol ceramide synthase |
| 0Z_04941 | GT32 | Mannosyltransferase or acetylglucosaminyltransferase |
| 0Z_00006 | GT39 | Dolichyl-phosphate-mannose--protein mannosyltransferase |
| 0Z_05203 | GT39 | Dolichyl-phosphate-mannose--protein mannosyltransferase |
| 0Z_05854 | GT39 | Dolichyl-phosphate-mannose--protein mannosyltransferase |
| 0Z_01730 | GT50 | Small COPII coat GTPase |
| 0Z_05696 | GT66 | Dolichyl-diphosphooligosaccharide--protein glycosyltransferase subunit |
| 0Z_08938 | GT69 | GDP-Man: α-1,3-mannosyltransferase (EC 2.4.1.-) |
| 0Z_00567 | CE1 | L-amino acid amidase |
| 0Z_01818 | CE10 | Probable kynurenine formamidase |
| 0Z_02938 | CE10 | Candidate carboxylesterase |
| 0Z_04185 | CE10 | Dipeptidyl-peptidase |
| 0Z_06406 | CE10 | Cholinesterase or sterol esterase |
| 0Z_06895 | CE10 | Crystal protein |
| 0Z_07757 | CE10 | Probable serine hydrolase |
| 0Z_01485 | CE16 | Thermolabile hemolysin |
| 0Z_00530 | CE4 | Chitin deacetylase |
| 0Z_02465 | CE4 | Chitin deacetylase |
| 0Z_04051 | CE4 | Chitin deacetylase |
| 0Z_04843 | CE4 | Acetyl xylan esterase or chitin deacetylase |
| 0Z_09402 | CBM13 | Previously known as cellulose-binding domain family XIII (CBD XIII) |
| 0Z_01051 | CBM19 | Modules of 60-70 residues with chitin-binding function |
| 0Z_09313 | CBM19 | Modules of 60-71 residues with chitin-binding function |
| 0Z_01395 | CBM20 | Alpha-amylase |
| 0Z_07796 | CBM32 | Anaphase-promoting complex subunit |
| 0Z_01416 | CBM50 | Also known as LysM domains |
| 0Z_02549 | CBM50 | Also known as LysM domains |
| 0Z_09154 | CBM50 | N-acetylmuramoyl-L-alanine amidase |
| 0Z_04487 | AA3 | Choline dehydrogenase |
| 0Z_09664 | AA3 | Glucose oxidase |
| 0Z_02627 | AA4 | D-lactate dehydrogenase |
| 0Z_07424 | AA5 | copper radical oxidase |
| 0Z_09029 | AA5 | Galactose oxidase |
| 0Z_09539 | AA5 | glyoxal oxidase precursor |
| 0Z_07169 | AA6 | NADH-quinone oxidoreductase |
| 0Z_00657 | AA7 | 6-hydroxy-D-nicotine oxidase |
| 0Z_08712 | AA7 | L-gulonolactone oxidase |
| 0Z_10770 | AA7 | 6-hydroxy-D-nicotine oxidase |
| 0Z_00380 | AA9 | Probable endo-beta-1,4-glucanase |
| 0Z_04190 | AA9 | endo-beta-1,4-glucanase |
| 0Z_04652 | AA9 | beta-1,4-endoglucanase |
| 0Z_08820 | AA9 | Cellulose-growth-specific protein |
| 0Z_09213 | AA9 | Endoglucanase-7 |
| 0Z_03104 | PL12 | heparinase II/III family protein |
| 0Z_02304 | PL14 | polysaccharide lyase |
| 0Z_03115 | PL15 | heparinase II/III family protein |

Note: Genes marked with grey colour were lignocellulolytic enzymes.
