# Supplementary material for: Investigation of lignocellulolytic enzymes during different growth phases of Ganoderma lucidum strain G0119 using genomic, transcriptomic and secretomic analyses
Source: PLoS One. 2018 May 31;13(5):e0198404. doi: 10.1371/journal.pone.0198404 (PMC5979026; doi:10.1371/journal.pone.0198404)
Supplement: S4 Table — (PDF) [file pone.0198404.s008.pdf]

Table S4. Key words for identification of the lignocellulolytic genes by annotation

Category	Gene name	CAZy family	Key words in annotation
Cellulase	Endo-beta-1,4-glucanase	GH5, GH9, GH12, GH45, GH74, GH131, CBM1, AA9	endoglucanase, endo-beta-1,4-glucanase, beta-,1,4-glucanase, xyloglucanase
	Exoglucanase	GH6, GH7, CE15	exoglucanase, cellobiohydrolase
	Beta-glucosidase	GH1, GH3	beta-glucosidase
Hemicellulase	Endo-1,4-beta-xylanase	GH10	endo-1,4-beta-xylanase
	Exo-1,4-beta-xylosidase	GH3	1,4-beta-xylosidase
	Beta-galactosidase	GH35, GH53	arabinogalactan, endo-1,4-beta-galactosidase, beta-galactosidase
	Mannosidase	GH2, GH38	alpha-mannosidase, beta-mannosidase
	Endo-1,5-alpha-L-arabinosidase	GH43	arabinan, endo-1,5-alpha-L-arabinosidase
	Alpha-L-arabinofuranosidases	GH51	alpha-L-rabinofuranosidase
Lignin-modifying enzyme	Laccase	AA1	laccase
	Manganese peroxidase	AA2	manganese peroxidase
	Versatile peroxidase	AA2	versatile peroxidase

Note: The lignocellulolytic genes were identified from the CAZy genes in the genome at first. Then, Key words were used for the identifying from the annotation of KEGG, NR and Uniprot. Genes met the given key words could be identified as putative lignocellulolytic genes.
